# Hyperprogressive disease in patients suffering from solid malignancies treated by immune checkpoint inhibitors: A systematic review and meta-analysis

**DOI:** 10.3389/fonc.2022.843707

**Published:** 2022-08-03

**Authors:** Zijun Zhao, Jin Bian, Junwei Zhang, Ting Zhang, Xin Lu

**Affiliations:** Department of Liver Surgery, Peking Union Medical College Hospital, Chinese Academy of Medical Sciences and Peking Union Medical College, Beijing, China

**Keywords:** hyperprogressive disease, solid malignancy, immune checkpoint inhibitor, incidence and prognosis, systematic review and meta-analysis

## Abstract

**Introduction:**

Hyperprogressive disease (HPD) is a paradoxically rapid disease progression during or shortly after antitumor treatment, especially immune checkpoint inhibitors (ICIs). Various diagnosis criteria of HPD cause heterogeneous incidence rates in different clinical research, and there is no consensus on potential risk factors associated with HPD occurrence. Hence, we aimed to summarize incidence of HPD in ICI treatment for solid tumors. Clinicopathological factors associated with HPD are also analyzed.

**Methods:**

Clinical studies about HPD during/after ICI treatment of solid malignancies are included. Pubmed, Embase, and Cochrane library were searched for eligible studies published before October 7. The Newcastle–Ottawa scale was used to assess the quality of the included studies. Random effect and fixed effect models were, respectively, used for pooling incidence of HPD and analysis of risk factors for HPD. Heterogeneity, subgroup analysis, and publication bias were also analyzed. All meta-analysis was performed *via* R software (y -40v4.0.2).

**Results:**

Forty-one studies with 6009 patients were included. The pooled incidence of HPD was 13.2% (95% CI, 11.2%–15.4%). Head and neck cancer (HNC) had the highest incidence of HPD (18.06%), and melanoma had the lowest (9.9%). Tumor types (*P* = .0248) and gender ratio (*P* = .0116) are sources of heterogeneity of pooled incidence of HPD. For five clinicopathological factors associated with HPD, only programmed cell death protein 1 ligand 1 (PD-L1) positivity was a preventive factor (odds ratio = 0.61, *P* <.05). High lactate dehydrogenase (LDH) level (OR = 1.51, *P* = .01), metastatic sites >2 (OR = 2.38, *P* <.0001), Eastern Cooperative Oncology Group Performance Score ≥2 (OR = 1.47, *P* = .02), and liver metastasis (OR = 3.06, *P* <.0001) indicate higher risk of HPD.

**Conclusions:**

The pooled incidence of HPD was less than 15%, and HNC had the highest incidence of HPD. LDH and PD-L1 are remarkable biomarkers for prediction of HPD in future medical practice.

## Introduction

Since the approval of immune checkpoint inhibitors (ICIs), such as programmed cell death protein 1 (PD-1) inhibitors and cytotoxic T lymphocyte–associated protein 4 (CTLA-4), the treatment blueprint of cancer has deeply altered, and researchers focus more on intervention of immune cells to make anticancer treatment more effective. By blocking the immunosuppressive receptors PD-1 and CTLA-4 on mature T cells, ICIs allow effector T cells to recognize and destroy cancer cells (1). Previous studies show an increase of overall survival (OS) in ICI treatment of various solid tumors, including endometrial stromal sarcoma, head and neck squamous cell carcinoma (HNSCC), melanoma, non-small cell lung cancer (NSCLC), renal cancer, and urothelial bladder cancer (2–4).

Nevertheless, some patients do not respond well to immunotherapy. Among them, some patients had a stable disease, whereas some patients suffer from disease progression with enlarged tumors or metastasis. In the latter part, the condition of some patients deteriorates with rapid acceleration of tumor growth during or after immunotherapy. This counterintuitive process with a dramatic disease progression is called hyperprogressive disease (HPD). HPD is an abnormal and drastic increment of tumor burden during or right after the initiation of ICI (5, 6). Before diagnosis of HPD, clinicians should differentiate HPD from pseudoprogression, a temporary enlargement of the tumor in radiological imaging caused by an inflammatory storm derived from the sudden infiltration of immune cells, followed by a stable antitumor response (5, 6).

Clinicians evaluate the response to immunotherapy for solid malignancies based on various guidelines, including response evaluation criteria in solid tumors (RECIST), immune-related response criteria (irRC), immune-related RECIST (irRECIST), immune RECIST (iRECIST), or immune-modified RECIST (imRECIST) (7–12). As a special type of tumor response, the diagnostic criteria of HPD were diversified according to different guidelines, and no consensus on a standardized definition of HPD exists. The most popular approach to HPD definition is derived from RECIST 1.1. HPD was first quantitively defined by Champiat et al. as “a progressive disease based on RECIST at initial evaluation as well as a 2-fold increment of tumor growth rate (TGR) during immunotherapy compared with baseline condition (pre-immunotherapy)” (5). For calculation of TGR, the calibration of tumor size is the prerequisite. According to RECIST 1.1, tumor size is referred to as the sum of the longest diameters of the target lesions (D) (7). If we presume the tumor to be a sphere, tumor volume can be approximated by V=πD^3^/6. Ideally, tumor growth is assumed to follow an exponential law in which *t* is set as the time in months when the tumor is evaluated, and the tumor volume at time *t* is expressed as V_t_ = V_0_exp^(TG.t)^ (TG is the growth rate of tumor; V_0_ stands for the tumor volume at baseline). Consequently, the formula of TG is TG = Log(D_t_/D_0_)/t (D_0_ and D_t_ stand for the tumor volume at baseline and time *t*, respectively). The term “TGR” is defined as the “percentage of increment of tumor volume during one month.” Hence, the formula of TGR is illustrated as TGR = 100 (exp(TG) - 1). According to Champiat’s research, HPD is calculated as TGR_exp_/TGR_ref_ ≥ 2 (TGR_exp_ stands for the TGR between baseline and first evaluation imaging; TGR_ref_ stands for the TGR between prebaseline and baseline). In this evaluation system, new lesions during treatment and a nonmeasurable focus at baseline are not included (5). Slightly different from the Champiat study, another French study published by Ferrara defines HPD as a more than 50% increase of TGR postimmunotherapy instead of the calculation of the TGR ratio (13). Some research teams use methods to evaluate tumor growth based on the combination of RECIST 1.1 and irRECIST. Compared with the definition of HPD in the Champiat study, a study led by Zhang added another two requirements: 1) time to treatment failure (TTF) < 2 months and 2) disease progression at the first evaluation and > 50% increase in TGR (14). In addition to TGR, other parameters were also created to recognize HPD. In the Saâda-Bouzid study, tumor growth kinetics (TGK) are defined as the difference of the sum of the largest diameters of the target lesions per unit of time. Thus, TGK_pre_ stands for the difference of the sum of the largest diameters of the target lesions per unit of time between prebaseline and baseline imaging, whose formula is (S_0_−S_PRE_)/(T_0_−T_PRE_); likewise, the formula of TGK_post_ is (S_post_−S_0_)/(T_post_−T_0_). Hyperprogression is defined as TGK_post_/TGK_pre_ ≥ 2 (15). In the Arasanz research, the approach to HPD definition is based on irRC (16). Its criteria of identification of HPD is similar to that of Champiat’s except that the identification of progressive disease is according to irRC instead of RECIST 1.1 (8). Regarding the study led by Kim et al., the definition of HPD uses the criteria of RECIST 1.1 and iRECIST. The definition of HPD is 1) TGK_post_/TGK_pre_ > 2 (the term TGK is the same as aforementioned); TTF < 2 months (17). In addition to TGR and TGK, some researchers define HPD by calculating the change of tumor burden before and after treatment. In a case series study led by Kato, four patients were evaluated as HPD because of a more than 50% increment of tumor burden postimmunotherapy (18). In the Mato, Petrova, and Lo Russo study, HPD is defined not only as a remarkable increase in tumor burden, but also the appearance of new lesions in distal organs (19–21). The different parameters of calculation of HPD are summarized in **Table 1**.

**Table 1:** Representative definition of hyperprogressive disease in different clinical studies.

	Champiat et al. (5)	Ferrara et al. (13)	Zhang et al. (14)	Saâda-Bouzid et al. (15)	Arasanz et al. (16)	Kim et al. (17)	Kato et al. (18)	Mato et al. (20)
**Assessment criteria**	RECIST 1.1	RECIST 1.1	RECIST 1.1; irRECIST	RECIST 1.1; irRECIST	irRC	RECIST 1.1; iRECIST	irRC	RECIST 1.1
**Parameter of tumor growth**	TGR	TGR	TGR	TGK	TGR	TGK	Tumor burden	Tumor burden
**Main formula**	TG = Log(D_t_/D_0_)/t TGR = 100 (exp(TG) - 1)	TG = Log(D_t_/D_0_)/t TGR = 100 (exp(TG) - 1)	TG = Log(D_t_/D_0_)/t TGR = 100 (exp(TG) - 1)	TGK = (S_t_−S_0_)/(T−T_0_)	TG = Log(D_t_/D_0_)/t TGR = 100 (exp(TG) - 1)	TGK = (S_t_−S_0_)/(T−T_0_)	Product of two-dimensional longest diameters of lesion	Longest diameter of lesion
**Definition of HPD**	TGR_exp_/TGR_ref_ ≥ 2 when PD (RECIST version) is eligible	PD according to RECIST on the first CT scan; (TGR_post_ - TGR_pre)_/TGR_pre_ ≥ 50%	TTF < 2 months; TGR_exp_/TGR_ref_ ≥ 1.5; ΔTGR/TGR_ref_ > 50%; PD at first evaluation after treatment	TGK_post_/TGK_pre_ ≥ 2	TGR_exp_/TGR_ref_ ≥ 2 when PD (irRC version) is eligible	TTF < 2 months; TGK_post_/TGK_pre_ > 2	TTF < 2 months; > 50% increase of tumor burden after immunotherapy; > 2-fold increase in progression pace	PD according to RECIST in the first 8 weeks after treatment initiation; Minimum increase in the measurable lesions > 10 mm (i) increase of ≥ 40% in sum of target lesions compared with baseline; and/or (ii) increase of ≥ 20% in sum of target lesions compared with baseline plus the appearance of new lesions in at least two different organs

D refers to sum of diameters of all targeted lesions; TGK_post_ stands for the difference of the sum of the largest diameters of the target lesions per unit of time between baseline and first-evaluation imaging; TGK_pre_ stands for the difference of the sum of the largest diameters of the target lesions per unit of time between prebaseline and baseline imaging; TGR_exp_ stands for TGR between baseline and first-evaluation imaging; TGR_ref_ stands for TGR between prebaseline and baseline.

iRECIST, immune RECIST; irRC, immune-related response criteria; irRECIST, immune-related RECIST; PD, progressive disease; RECIST, Response evaluation criteria in Solid Tumors; TG, velocity of tumor growth; TGK, tumor growth kinetics; TGR, tumor growth rate; TTF, time-to-treatment failure.

HPD is reported in various solid malignancies, including NSCLC, gynecologic malignancies, urologic tumors, melanoma, hepatocellular carcinoma, and digestive system cancers (22–28). Compared with patients suffering from natural progressive disease (PD), patients suffering from HPD have worse symptoms, poor performance status, and shorter OS as well as progression-free survival (PFS) (5, 13, 29). Worse is that available salvage treatment is limited (30). With the rapid expansion of application of ICIs in different solid malignancies, the phenomenon of this accelerated progression seems to be inevitable in clinical experience. Currently, the majority of studies about HPD are small-scale retrospective studies or case reports. Incidence, predictors, and prognosis of HPD in each type of solid malignancy are not well-elucidated. Moreover, the varieties of diagnostic criteria of HPD make it difficult for scientists to compare incidence of HPD in different tumors. Hence, we performed this systematic review and meta-analysis to summarize the major clinical characteristics of HPD, definitions and incidence of HPD, associated risk predictors, and clinical outcomes of HPD.

## Methods

Our study strictly followed the Preferred Reporting Items for Systematic Reviews and Meta-Analysis statement.

### Literature search and selection

A comprehensive search in the databases of Pubmed, Embase, and Cochrane library was performed to collect eligible studies before October 7, 2021. Two researchers (ZJZ and XL) independently conducted the literature search and data extraction. Key terms used for literature mining included “hyperprogressive,” “hyperprogression,” “hyperprogressor,” and “hyperprogressive disease.” Language of papers was restricted to English. Studies were reviewed and selected base on titles, abstracts, and full texts sequentially.

According to the question-based PICOS approach, our inclusion criteria included 1) population with solid malignancies; 2) main intervention is ICI agents [PD-1, Programmed cell death protein 1 ligand 1 (PD-L1), CTLA-4 inhibitor]; 3) primary outcome is incidence of HPD, and secondary outcome is survival; and 4) prospective or retrospective studies. The exclusion criteria included 1) duplication; 2) non-English articles; 3) other types of publication: basic studies, case reports, conference abstracts, news, comments, editorials, letters, reviews, systematic review and meta-analysis and other special types of literatures (notes, etc.); 4) subject of studies were related to hematological malignancies and other non–solid tumor diseases; and 5) studies not related to immunotherapy. Studies failing to meet the inclusion criteria were excluded. Disagreement between the two reviewers were solved by discussion.

### Data extraction and quality evaluation

Two authors (ZJZ and XL) independently performed data extraction from eligible studies and assessed the risk of bias. The following data were collected: first author, year of publication, sample size, number of patients who suffered from HPD, age (median/mean), gender ratio (male:female), study design (prospective/retrospective), disease, immunotherapeutic agents, median OS, median PFS; risk factors, HPD criteria. A tree diagram was illustrated to extract and summarize the main approaches to defining HPD. Quality assessment was conducted based on the Newcastle-Ottawa scale (NOS), which is a practical instrument for quality assessment of nonrandomized studies. The total score of the NOS scale is nine points, which is dispensed in three models with a total of eight questions: selection (maximum of four points), comparability (maximum of two points)m and outcomes (maximum of three points) for cohort studies (31).

### Statistical analysis

R version 4.0.2 was used to complete the whole process of meta-analysis. Statistical heterogeneity was tested by the Higgins inconsistency index (I^2^) test. If the statistic I^2^ was less than 50% and the *P*-value was greater than.1, incidence of HPD in these studies is recognized as homogenous, and the integrated incidence is calculated by a fixed effect model, or a random effect model (the DerSimonian–Laird method) is used. For meta-analysis of binary outcome data, function “metabin ()” was used in which the summary measure is odds ratio (OR). For meta-analysis of single proportions, function “metaprop ()” was used and Logit transformation of the raw data was prepared for the following data processing. Publication bias was assessed by funnel plots and Egger’s tests. Subgroup analysis was conducted to calculate pooled incidence of HPD according to different tumor types, different definitions of HPD, different ethnicities and different gender ratio (male:female). In order to examine the robustness of the results of our analysis, sensitivity tests were performed to calculate the pooled incidence after each one of the 44 studies was excluded (the so-called “leave-one-out” method). All the tests above were two-tailed, and *P* <.05 was considered statistically significant except for the aforementioned test of heterogeneity.

## Results

**Figure 1:**
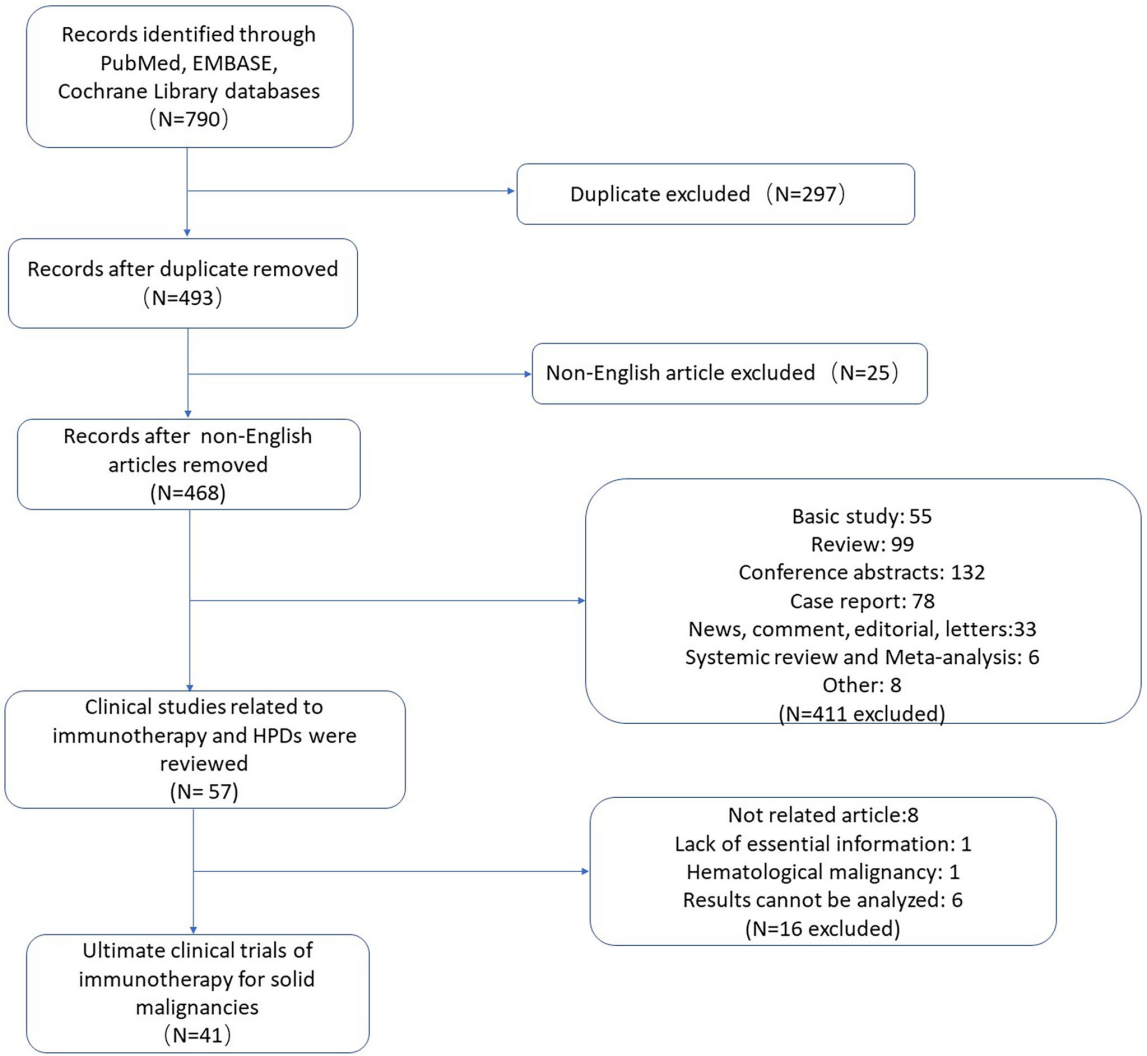
Flow chart of literature selection process.

**Figure 1** illustrates the whole process of study selection. A total of 790 literatures were collected from the databases of Pubmed, Embase, and Cochrane Library. The specific search strategy is shown in **Supplementary Table 1**. After excluding duplicate studies, non-English studies, and studies other than clinical research (basic study, case report, review, letter, editorial, conference abstract, systematic review, and meta-analysis, etc.), 57 studies were then selected for further screening. Among them, the content of eight studies were not related to ICI treatment of solid malignancies, one study was about ICI treatment of hematological malignancies, and one study had a shortage of some essential information for further meta-analysis. Another six studies were hard to integrate with other studies for further analysis. Ultimately, 41 studies are listed in this meta-analysis **(** **Table 2** **)**.

**Table 2:** Characteristics of finally included studies and patients.

Author	Country	Year	*N* (total)	*N* (HPD)%	Age (median/mean, range/ ± SD)	Gender ratio(male: female)	Study design	Disease	ICI agent	Risk factors	Definition of HPD	HPD criteria	Reference
Yilmaz, M.	Turkey	2021	54	7 (13.0%)	62.3^*^ (25–85)	16:11	R	Metastatic melanoma; Renal cell carcinoma	Nivolumab	NA	RECIST progression+ at least 3 of: 1. TTF<2months; 2. ≥50% increase of sum of target lesions major diameter; 3. ≥2 new lesions in organ already involved or spread to a new organ 4. ECOG PS≥2	iRECIST	(32)
Takahashi, Y.	Japan	2021	487	45 (9.2%)	70^#^ (26–90)	347:140	P	Gastric cancer	Nivolumab	Peritoneal metastasis	TGR_post_/TGR_pre_≥2	RECIST 1.1	(33)
Rocha, P.	Spain	2021	42	6 (14.3%)	67.5^#^ (50–86)	6:1	R	NSCLC	Nivolumab	NA	TGR_post_/TGR_pre_≥2; TGK_post_/TGK_pre_≥2; 40% increase compared with the baseline sum of the target lesions; an increase of 20% in the sum of target lesions and the appearance of new lesions in at least two different organs.	RECIST 1.1	(34)
Schuiveling, M.	Netherland	2021	162	2 (1.2%, including possible HPD)	NA	11:7	R	Melanoma	Anti-PD1 Anti-CTLA4	NA	If no pre-baseline scan was available TTF<2 months + 2×volumetric tumor burden in 2 months If pre-baseline scan was available TGR_post_/TGR_pre_≥2	RECIST 1.1; iRECIST 1.1	(35)
Nakamoto, R.	US	2021	76	9 (11.8%)	60.5 (48.5–75, IQR)	49:27	R	Malignant Melanoma	Ipilimumab Pembrolizumab Nivolumab		TGK_post_/TGK_pre_≥2	RECIST 1.1; irRECIST	(36)
Zhang, L.	China	2021	69	10* (14.5)	42	9:1	R	HCC	Nivolumab Pembrolizumab Camrelizumab	Hemoglobin level; Portal vein tumor thrombus; Child-Pugh score	(1) TTF<2 months; (2) disease progression at the first evaluation and > 50% increase in TGR; (3) TGR_post_/TGR_pre_≥2	RECIST 1.1; irRECIST	(14)
Jin, T.	China	2021	67	17 (25.4%)	NA	50:17	R	NPC	anti-PD-1 mAb;	Elevating LDH, high metastatic burden, liver metastasis	(1) the acceleration of tumor cells proliferation exceeding twice as much or as many based on three point of time (pre-treatment, baseline, post/under-treatment) (2) TTF ≤ 2 months	RECIST 1.1	(37)
Economopoulou	Greece	2021	117	18* (15.4)	62^#^ (40–88)	NA	R	HNSCC	anti-PD-1 mAb; anti-PD-L1 mAb; anti-CTLA4 mAb	Younger age; primary tumor in oral cavity; Second/third-line treatment of ICI treatment	TGK_post_/TGK_pre_≥2	RECIST 1.1; irRECIST	(38)
Kim	Korea	2021	231	26 (11.3)	58.96 ± 10.2*	23:3	R	NSCLC	nivolumab, pembrolizumab, avelumab, atezolizumab, durvalumab	smoking ≥20 pack.Years; PD-L1 expression ≤1%; the presence of oncogenic driver mutation; Number of metastatic sites ≥3	TGK_post_/TGK_pre_≥2 TTF ≤ 2 months	RECIST 1.1; iRECIST	(17)
Kim, C. G.	Korea	2021	189	24 (12.7%)	62 (28-82)	92:21	R	HCC	Nivolumab	Elevated neutrophil-to-lymphocyte ratio	TGK_post_/TGK_pre_≥4 TGR_post_/TGR_pre_≥4 TTF ≤ 2 months >40% increase in ΔTGR	RECIST 1.1	(39)
Choi, W. M.	Korea	2021	194	18 (9.3%)	57.4^*^ ± 11.3	159:35	R	HCC	Nivolumab	Large ΔNLR at 4 weeks	TGK_post_/TGK_pre_≥4 TGR_post_/TGR_pre_≥4	RECIST 1.1	(40)
Chen	China	2021	377	38 (10.1%)	NA	NA	R	Multiple cancers	Nivolumab Pembrolizumab	more metastatic sites Liver metastasis, ECOG score ≥ 2, Elevation of LDH before immunotherapy; KRAS status	(TGR_post_ - TGR_pre)_/TGR_pre_≥50%	RECIST 1.1	(41)
Ayers, K. L.	US	2021	249	41 (16.5%)	67.3^#^ (61.3–74.3, IQR)	127:122	R	NSCLC	Nivolumab, Pembrolizumab, Atezolizumab	NA	TTF <2 months	NA	(42)
Castello, A.	Italy	2020	50	14 (30.4%, 14/46)	73^#^	17:8	P	NSCLC	Nivolumab, Pembrolizumab, Atezolizumab	NA	PD on the first CT scan according to RECIST 1.1 (TGR_post_ - TGR_pre)_/TGR_pre_≥50%	iRECIST	(43)
Choi	Korea	2020	78	15 (19.2%)	61.3^*^ ± 11.3	49:29	R	NSCLC	Nivolumab, Pembrolizumab, Atezolizumab	Younger age; larger primary lesion size; greater number of metastatic sites	Fulfilling 3 of 5 of below: 1. TTF<2months; 2. ≥50% increase of sum of target lesions major diameter between baseline and the first radiologic evaluation; 3. ≥2 new lesions in organ already involved or spread to a new organ between baseline and the first radiologic evaluation; 4. appearance of a new organ lesion between baseline and the first radiologic evaluation; 5. Decrease of ECOG PS≥2 during the first two months of treatment	RECIST 1.1	(44)
Hagi, T.	Japan	2020	136	30 (22.6%)	NA	110:33	R	Gastric cancer	Nivolumab	Liver metastasis, PS score 2-3	> 50% increase in the sum of the longest diameter of the target lesions TGK_post_/TGK_pre_≥2	RECIST 1.1	(45)
Hwang, I.	Korea	2020	203	13 (6.4%)	64^#^ (56-71)	141:62	R	Renal cell carcinoma; urothelial carcinoma	anti-PD-1 mAb; anti-PD-L1 mAb	Age≥65; Urothelial carcinoma; Cr>1.2 mg/dL; Liver metastasis; >2 sites of metastases; <30% increase of lymphocyte	TGR_post_/TGR_pre_≥2 TTF ≤ 2 months > 50% increase in the sum of the longest diameter of the target lesions	RECIST 1.1	(46)
Petrova	Bulgaria	2020	167	16 (10.0)	60·2^*^ ± 6·8	1:1	R	NSCLC	Pembrolizumab	High level of neutrophil- lymphocyte ratio; presence of sarcopenia	Fulfilling 3 of 5 of below: 1. TTF<2 months; 2. ≥50% increase of sum of target lesions major diameter between baseline and the first radiologic evaluation; 3. ≥2 new lesions in organ already involved or spread to a new organ between baseline and the first radiologic evaluation; 4. appearance of a new organ lesion between baseline and the first radiologic evaluation; 5. Decrease of ECOG PS≥2 during the first two months of treatment	RECIST 1.1	(21)
Petrioli, R.	Italy	2020	47	3 (6.4%)	68 (44-82)	34:13	R	Multiple tumor	Nivolumab	High metastatic burden; High LDH and NLR	TGR_post_/TGR_pre_≥2	RECIST criteria 1.1	(47)
Park, J. H.	Korea	2020	125	18 (14.4%)	57 (33–87)	103:22	R	HNSCC	anti-PD-1 mAb; anti-PD-L1 mAb; anti-CTLA4 mAb	Younger age, Oropharyngeal cancer, Prior radiotherapy to locoregional lesion	TGK_post_/TGK_pre_≥2	RECIST 1.1	(48)
Okamoto, I.	Japan	2020	52	8 (15.4%)	65^#^ (28–81)	45:7	R	HNC	Nivolumab	PD-L1 expression <40%	PD according to RECIST 1.1 TGR_post_/TGR_pre_≥2	RECIST 1.1	(49)
Refae, S.	France	2020	98	11 (11.2%)	68^#^ (32–85)	65:33	R	Multiple tumors	anti-PD-1 mAb; anti-PD-L1 mAb	age ≥ 70 years, VEGFR2, PDL1	TGK_post_/TGK_pre_≥2	RECIST 1.1	(50)
Ruiz-Patiño, A.	Columbia	2020	296	44 (14.9%)	64 (34–90)	177:119	R	NSCLC	Ipilimumab, Nivolumab, Pembrolizumab, Dvalumab, Avelumab	NA	NA	RECIST	(51)
Karabajakian, A.	France	2020	120	22 (18.3%)	NA	97:23	R	HNSCC	anti-PD-1 mAb; anti-PD-L1 mAb	Higher NLR	TGK_post_/TGK_pre_≥2	RECIST 1.1	(52)
Forschner, A.	Germany	2020	51	22 (43.1%)	71 (40-87)	9:8	R	Melanoma	Nivolumab, Pembrolizumab, Ipilimumab	MDM2/4 or EGFR amplification or <1% PD-L1 positive tumor cells	PD according to RECIST > 50% increase in the sum of the total measured tumor burden	RECIST 1.1	(53)
Arasanz	Spain	2020	56	10 (17.9)	NA	26:9	P	NSCLC	Atezolizumab Nivolumab Pembrolizumab	Smoking; Expansion of CD28- CD4 lymphocytes	PD according to RECIST at first CT scan TGR_post_/TGR_pre_≥2	RECIST 1.1; irRC	(16)
Matos	Spain	2020	270	29 (10.7)^1^		121:149	P	Multiple solid tumors	anti-PD-1 mAb; anti-PD-L1 mAb	Presence of liver metastasis; Having more than two metastatic sites before treatment with ICIs	PD according to RECIST in the first 8 weeks after treatment initiation; Minimum increase in the measurable lesions > 10mm (i) increase of ≥40% in sum of target lesions compared with baseline; and/or (ii) increase of ≥20% in sum of target lesions compared with baseline plus the appearance of new lesions in at least two different organs	RECIST 1.1	(20)
Kim, S. H.	Korea	2020	83	16 (19.3%)	60 (53, 68)	55:28	R	NSCLC	Nivolumab	Pleura or pericardium metastasis, Increased effusion	PD according to RECIST at first evaluation TGR_post_/TGR_pre_≥2	RECIST 1.1	(54)
Lau	China	2020	50	5 (10.0)	64^#^ (22-87)	33:17	R	Multiple cancer	Pembrolizumab Nivolumab	NA	NA	RECIST 1.1	(55)
Lu, Z.	China	2019	56	5 (8.9%)	NA (22-77)	39:17	R	GI tract cancer	anti-PD-1 mAb; anti-PD-L1 mAb; anti-CTLA4 mAb	Concentrations of MCP-1, LIF, PD-L2, IL-21, CD152	NA	RECIST 1.1	(56)
Aoki	Japan	2019	34	10 (29.4)	67^#^ (51–84)	13:4	R	Gastric cancer	Nivolumab	NA	TGR_post_/TGR_pre_≥2	RECIST 1.1	(57)
Kanjanapan	Canada	2019	182	12 (7.0)	60 (21-89)	83:88	R	Multiple cancers	anti-PD-1 mAb; anti-PD-L1 mAb; anti-CTLA4 mAb	Female sex	PD according to RECIST at first evaluation TGR_post_/TGR_pre_≥2	RECIST 1.1	(58)
Kim, C. G.	Korea	2019	263	54 (20.5%)	63^#^ (26–85)	191:72	R	NSCLC	anti-PD-1 mAb; anti-PD-L1 mAb	Larger metastatic burden, liver metastasis, LDH elevation	TTF<2 months TGR_post_/TGR_pre_≥2 Or TGK_post_/TGK_pre_≥2	RECIST 1.1	(29)
Kim, Y.	Korea	2019	335	44 (13.1%)^1^	NA	94:41	R	NSCLC	Nivolumab, Pembrolizumab, Atezolizumab, Durvalumab, Avelumab	NLR≥4, LDH≥ULN	TTF<2 months TGK_post_/TGK_pre_≥2 > 50% increase in the sum of the total measured tumor burden	RECIST 1.1	(59)
Sasaki, A.	Japan	2019	62	13 (21.0%)	67 (25–86)	45:17	R	Gastric cancer	Nivolumab	High ECOG score, liver metastasis, Larger tumor size at baseline	TGK_post_/TGK_pre_≥2 > 50% increase in the sum of the total measured tumor burden	RECIST v1.1	(60)
Scheiner, B.	Austria/ Germay	2019	65	4 (6.2%)	65.2^*^ ± 11.1	49:16	R	HCC	Nivolumab Pembrolizumab	NA	PD on the first CT scan according to RECIST 1.1 (TGR_post_ - TGR_pre)_/TGR_pre_≥50%	mRECIST; RECIST 1.1	(61)
Tunali, I.	US	2019	187	15		105:82	P	NSCLC	Nivolumab, Pembrolizumab, Durvalumab, Atezolizumab, Ipilimumab, Tremelimumab		TTF<2 months TGR_post_/TGR_pre_≥2 PD on the first CT scan according to RECIST 1.1	RECIST 1.1; iRECIST	(62)
Ten Berge	Netherlands	2019	58	4 (6.9%)	64^#^ (35–79)	17:12	R	NSCLC	Nivolumab	No risk factors	TGR_post_/TGR_pre_≥2	RECIST version 1.1	(63)
Ferrara	France	2018	406	56 (13.8%)	NA	NA	R	NSCLC	Nivolumab, Pembrolizumab, Atezolizumab, Durvalumab	More than 2 metastatic sites	PD according to RECIST on the first CT scan; (TGR_post_ - TGR_pre)_/TGR_pre_≥50%	RECIST 1.1	(13)
Saâda-Bouzid	France	2017	34	10 (29.4)	63	8:2	R	HNSCC	anti-PD-1 mAb; anti-PD-L1 mAb	Presence of a regional recurrence	TGK_post_/TGK_pre_≥2	RECIST 1.1; irRECIST	(15)
Champiat^△^	France	2017	131	12 (9.2%)	65.6^#^ (32-82)	4:8	P	Multiple cancers	anti-PD-1 mAb; anti-PD-L1 mAb	Older age	TGR_post_/TGR_pre_≥2 PD on the first evaluation according to RECIST 1.1	RECIST 1.1	(5)

*mean.

^#^median.

P, Prospective study; R, retrospective study.

^1^HPD definition based on RECIST 1.1 criteria.

CD152, cluster of differentiation 152; CTLA4, cytotoxic T-lymphocyte associated protein 4; ECOG PS, Eastern Cooperative Oncology Group Performance Score; EGFR, epidermal growth factor receptor; GI tract, Gastrointestinal tract; HCC, hepatocellular carcinoma; HNC, head and neck cancer; HNSCC, head and neck squamous cell carcinoma; HPD, hyper-progressive disease; ICI, immune checkpoint inhibitor; IL-21, interleukin 21; IQR, interquartile range; iRECIST, immune RECIST; irRC, immune-related response criteria; LDH, lactate dehydrogenase; LIF, leukocyte inhibition factor; mAb, monoclonal antibody; MCP-1, monocyte chemotactic protein 1; MDM2/4, murine double minute2/4; NA, not available; NLR, neutrophil-to-lymphocyte ratio; NPC, nasopharyngeal carcinoma; PD, progressive disease; PD-1, programmed cell death 1; PD-L1 programmed cell death ligand 1; PD-L2, programmed cell death ligand 2; TGK, tumor growth kinetics; TGR, tumor growth rate; TTF, time to treatment failure; ULN, upper limit of normal; VEGFR2, Vascular Endothelial Growth Factor Receptor 2 gene.

All 41 studies were evaluated by the NOS scale, and the score ranged from four to nine with a median score of seven **(** **Supplementary Table 2** **)**. In total 24 studies were marked as higher than or equal to seven points. In a selection of cohort model, six of 41 studies were awarded four stars, and the other 35 were offered 35 stars. Twenty-nine studies were awarded in evaluation of comparability, among which 19 studies were awarded two stars, and the other 10 got only one star. Regarding the outcome model, 14 studies were given one point, and the other 27 studies were given three points.

Information on each eligible study is summarized in **Table 2**. In total, 6009 patents in 41 clinical studies were included. Six prospective studies and 35 retrospective studies were published between 2017 and 2021. Major types of diseases included NSCLC (14 studies), melanoma (three studies), head and neck cancers (HNC in six studies including HNSCC and NPC), hepatocellular carcinoma (HCC in four studies), gastrointestinal cancers (five studies). Another nine studies included multiple types of cancers. The sample size of each study ranged from 34 to 487, and the male population outnumbered the female counterpart in the majority of studies except four of them. In the Petrova study, the population of both genders were distributed equally, whereas in the studies of Matos, Kanjanapan, and Champiat, the female population was larger than the male counterpart. Thirty studies reported the average or median age of the whole cohort. The mean/median age ranged from 42 to 73 years. Immunotherapy was limited to ICIs, and the most popular types in all 41 studies included PD-1 inhibitors (Nivolumab, Pembrolizumab, Camrelizumab), PD-L1 inhibitors (Avelumab, Atezolizumab, Durvalumab), and CTLA-4 inhibitors (Ipilimumab, Tremelimumab). In some studies, the names of the ICI agents were not available.

**Figure 2:**
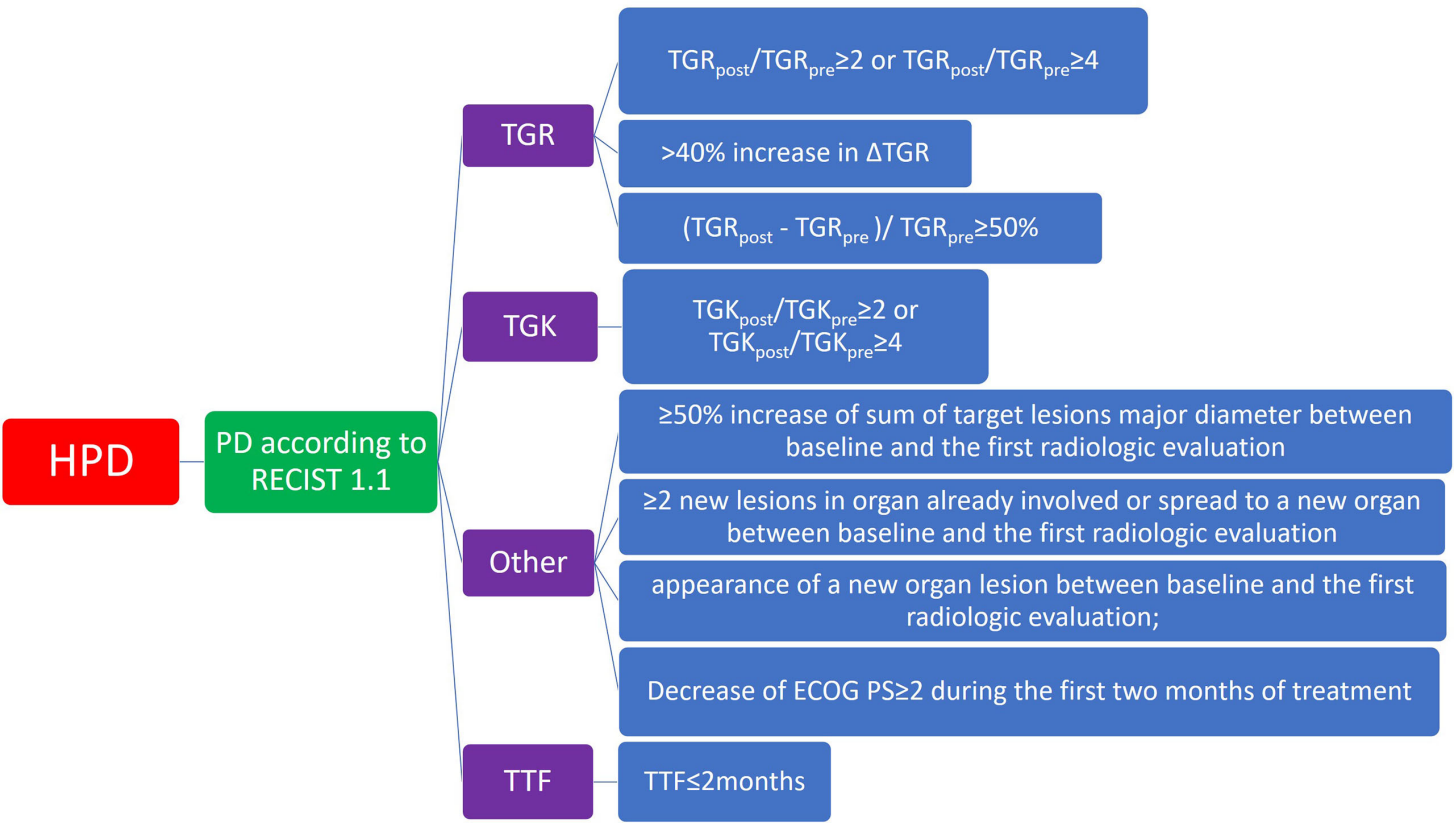
Diagram of category of major definition of hyperprogressive diseases in included studies. ECOG PS, Eastern Cooperative Oncology Group Performance Score; HPD, hyperprogressive disease; PD, progressive disease; RECIST, Response evaluation criteria in Solid Tumors; TGK, tumor growth kinetics; TGR, tumor growth rate; TTF, time to treatment failure.

Speaking of the definition of HPD, different studies recognized discrepant combinations of several HPD criteria. Thus, no consensus was put forward to best describe the feature of this ICI-related complication. Currently, the whole system of HPD criteria can be illustrated as a categorized tree diagram as showed in **Figure 2**. No matter which kind of definition of HPD, the general prerequisite is a confirmed progressive disease based on RECIST evaluation. It is necessary to differentiate from pseudoprogression before the determination of HPD. Based on this assurance, the description of HPD is mainly dependent on several parameters: TGR, TGK, and TTF. Definitions of these terms are mentioned in “methods.” TTF refers to the duration between the beginning of immunotherapy to drug discontinuation for any reason, including toxicity, disease progression, and even death (64). The TGR-dependent definition of HPD in eligible studies included “TGR_post_/TGR_pre_ ≥ 2” or “TGR_post_/TGR_pre_ ≥ 4,” whereas certain studies define HPD as “(TGR_post_ - TGR_pre)_/TGR_pre_ ≥ 50%” or “> 40% increase in ΔTGR.” For the TGK-dependent definition of HPD, popular criteria were “TGK_post_/TGK_pre_ ≥ 2” or “TGK_post_/TGK_pre_ ≥ 4.” Whatever the usage of TGR or TGK, most studies add the TTF ≤ 2 because rapid progression is an essential trait of HPD. Different from traditional TGK and TGR, some researchers use other approaches to defining HPD. These methods consist of “≥ 50% increase of sum of target lesion major diameter between baseline and the first radiologic evaluation,” “≥ 2 new lesions in organ already involved or spread to a new organ between baseline and the first radiologic evaluation,” “appearance of a new organ lesion between baseline and the first radiologic evaluation,” and “decrease of Eastern Cooperative Oncology Group Performance Score (ECOG PS) ≥ 2 during the first two months of treatment.” These definitions create different results according to different types of tumor response criteria, such as RECIST, irRC, irRECIST, iRECIST, or immune-modified RECIST.

Regarding clinical outcomes, 20 of 41 studies and 15 of 41studies report the OS and PFS of the whole population, respectively. One study led by Sasaki failed to reach the median OS. For all subjects, median OS ranged from 3.9 to 19 months, whereas median PFS was between 1.4 and 16.8 months. Twenty-six studies analyze the OS of HPD, non-HPD, or non-HPD PD patients. For the non-HPD cohort, OS ranged from 5.5 to more than 60 months, non-HPD PD patients had OS between 2.4 and 25 months, and the HPD cohort between 1.67 and 9.83 months. For comparison between HPD and non-HPD cohorts, 15 of 17 studies found that the OS of HPD patients was significantly shorter than that of non-HPD patients, whereas the Kanjanapan and Saâda-Bouzid studies failed to show a significant difference of OS between the two groups. When comparing HPD and a natural PD cohort, 10 of 16 studies found the median OS of HPD patients was significantly shorter than the other cohort, whereas the other six studies did not find a remarkable difference. Thirteen of 41 studies reported median PFS of non-HPD, non-HPD PD, or HPD cohorts. The median PFS of non-HPD patients was between 1.7 and 6.1 months. Patients with natural PD had a median PFS ranging from 0.8 to 1.7 months and HPD patients between 0.43 to 1.9 months. Nine studies compared median PFS between non-HPD and HPD patients, and all of these studies found that median PFS of HPD patients was significantly lower than that of non-HPD patients. In seven studies reporting a comparison between HPD and non-HPD PD patients, five studies demonstrated a significantly lower median PFS in the HPD cohort, whereas the other two studies (the Aoki and Park studies) did not find a discrepancy between these two cohorts **(** **Table 3** **)**.

**Table 3:** Data of survival analysis in 41 included studies.

Author	Year	Median follow-up time (months)	Median OS (months)				HR and P-value of OS		Median PFS (months)				HR and P-value of PFS		Reference
			Whole cohort	Non-HPD	HPD	Non-HPD PD	HPD vs. Non-HPD	HPD vs. Non- HPD PD	Whole cohort	Non-HPD	HPD	Non-HPD PD	HPD vs. Non-HPD	HPD vs. Non- HPD PD	
Yilmaz, M.	2021		NA						NA						(32)
Takahashi, Y.	2021		5.82	5.72	2.79	2.40	1.77, *P* = .001	1.05, *P* = .8	1.84						(33)
Rocha, P.	2021		13.3		5	6.7			4.8						(34)
Schuiveling, M.	2021		NA						NA						(35)
Nakamoto, R.	2021	19		>60	7	25	*P* = .0001	*P* = .01							(36)
Lu Zhang	2021		7.9	10.3	6		4.79, *P* <.001	2.50, *P* = .05		NA					(14)
Jin, T.	2021	7		NA						NA					(37)
Economopoulou	2021	10.7	10.7	15	6.53		*P* = .0018		2.8	6.1	1.8		*P* = .0001		(38)
Kim	2021			NA						NA					(17)
Kim, C. G.	2021			NA	59 days	96 days		2.238 *P* <.001	NA		23 days	48 days		2.194 *P* <.001	(39)
Choi, W. M.	2021			NA			2.25			NA					(40)
Chen	2021		NA		3.6	7.3		*P* <.01	NA						(41)
Ayers, K. L.	2021		17.4							NA					(42)
Castello, A.	2020		12.3	15.2	4		*P* = .003			NA					(43)
Choi	2020		12	13	4	6	*P* = .021	*P* = .002		NA					(44)
Hagi, T.	2020		7.6		3.3	6.8		*P* = .012	2.2		1.2	1.7		*P* <.001	(45)
Hwang, I.	2020	15.2		NA	3.5	7.3		*P* <.001		3.9	1.3		*P* <.001		(46)
Petrova	2020		NA		9.83	17.32		*P* < ·001		NA					(21)
Petrioli, R.	2020	11.8	12.3						6.2						(47)
Park, J. H.	2020	12.1	10.8	10.7	5.0		*P* = .047	*P* = .416	2.7	3.4	1.2		*P* <.001	*P* = .172	(48)
Okamoto, I.	2020		9.6						4.0						(49)
Refae, S.	2020	13.3		Not reached				*P* = .003	16.8						(50)
Ruiz-Patiño, A.	2020		12.7						4.27						(51)
Karabajakian, A.	2020			14.6	3.8		2.2, *P* = .0018			3.9	1.9		2.8, *P* <.0001		(52)
Forschner, A.	2020		19							NA					(53)
Arasanz	2020		48.1 weeks	54.7 weeks	14 weeks		*P* = .006		8.9 weeks	10.9 weeks	6 weeks		*P* <.001	*P* = .044	(16)
Matos	2020			7.33	5.23		1.73 *P* = .04			NA					(20)
Kim, S. H.	2020		8.6		2.33	4.11		1.18 *P* = .641	2.6		0.43	1.35		3.654 *P* = .001	(54)
Lau	2020			NA						NA					(55)
Lu, Z.	2019			11.4	3.6		*P* <.01			4.2	1.4		*P* <.001		(56)
Aoki	2019		3.9	5.5	2.1	3.1	4.7 *P* = .00195	2.1 *P* = .168	1.4	1.7	0.9	0.8	3.4 *P* = .00426	1.1 *P* = .756	(57)
Kanjanapan	2019			14.3	5.9		1.7 *P* = .11			2.8	1.6		3.7 *P* <.001		(58)
Kim, C. G.	2019				50 days	205 days		5.079 *P* <.001		NA	19 days	48 days		4.619 *P* <.001	(29)
Kim, Y.	2019			7.9	4.7		*P* =.009			NA					(59)
Sasaki, A.	2019		Not reached	Not reached	2.3		9.2 *P* <.001		2.0	2.4	0.7		4.8 *P* <.001		(60)
Scheiner, B.	2019		11.0						4.6						(61)
Tunali, I.	2019		NA							NA					(62)
Ten Berge, Dmhj	2019		11.5 ± 2.8	12.3 ± 4.3	2.3 ± 2.7		*P* = .041			NA					(63)
Ferrara	2018	12.1	13.4		3.4	6.2		2.18 *P* = .003	2.1						(13)
Saâda-Bouzid	2017	10.3	8	8.1	6.1		*P* = .77								(15)
Champiat^△^	2017				4.6	7.6		*P* = .19		NA					(5)

HPD, hyperprogressive disease; HR, hazard ratio; NA, not available; OS, overall survival; PD, progressive disease; PFS, progression-free survival.

**Figure 3:**
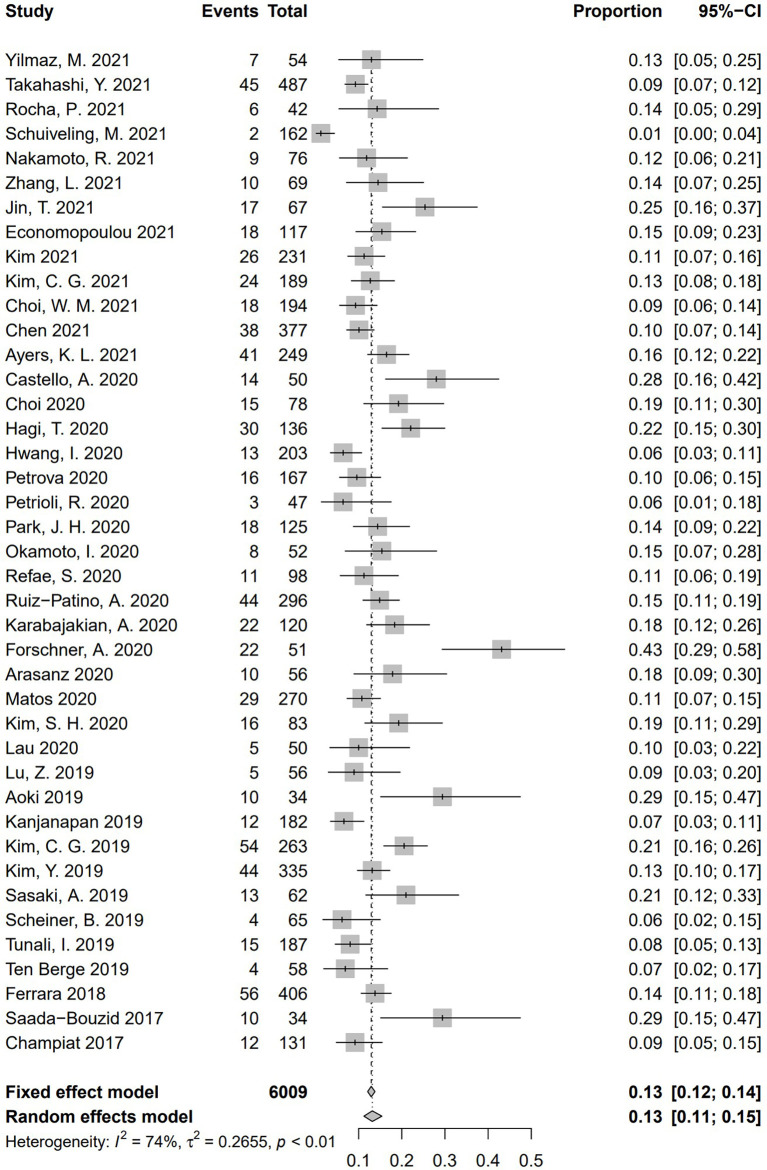
Forest plots of overall pooled incidence of hyperprogressive disease. CI, confidence interval.

**Figure 4:**
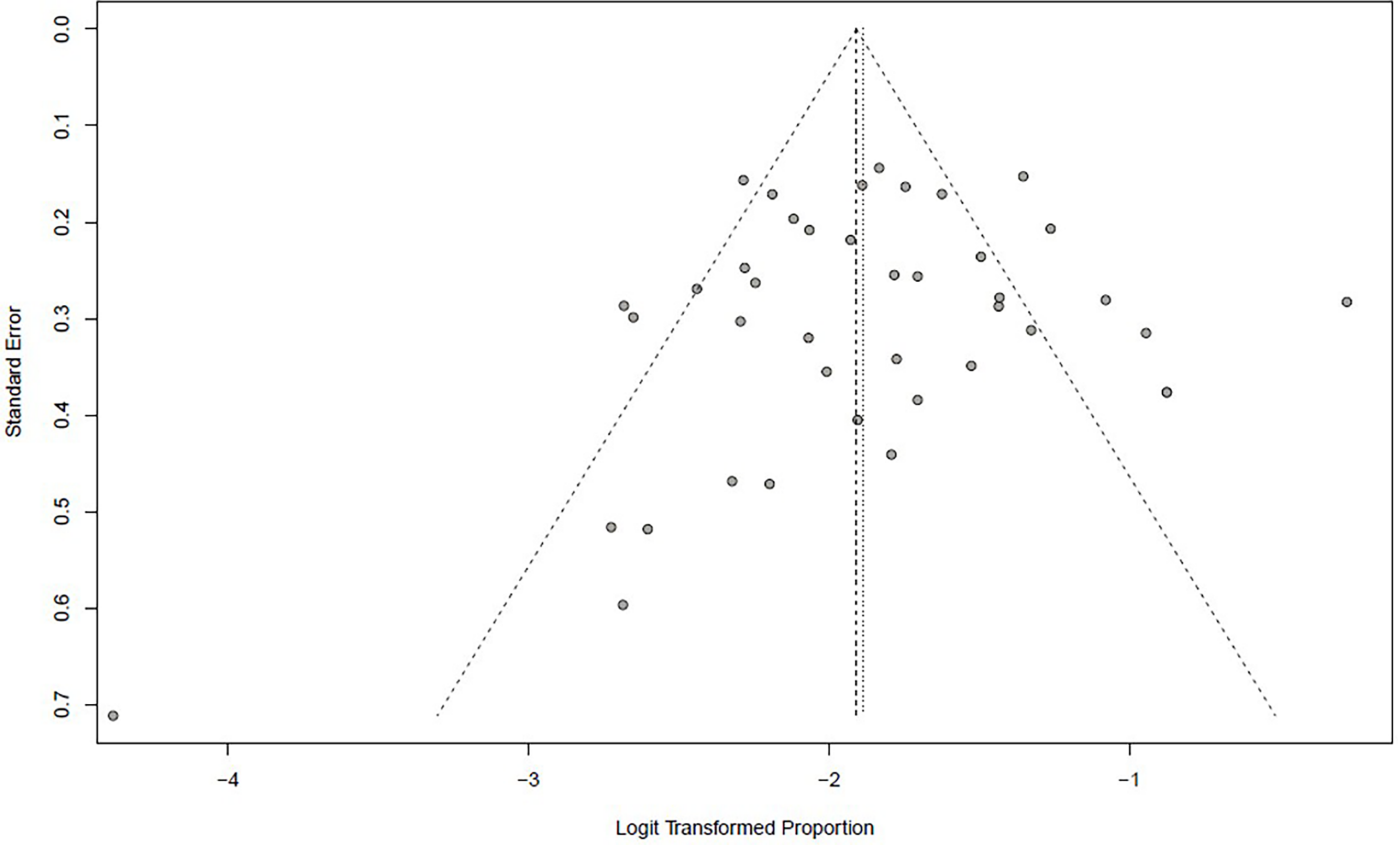
Funnel plot illustrating the publication bias of meta-analysis of overall pooled incidence rate of hyperprogressive disease.

**Figure 5:**
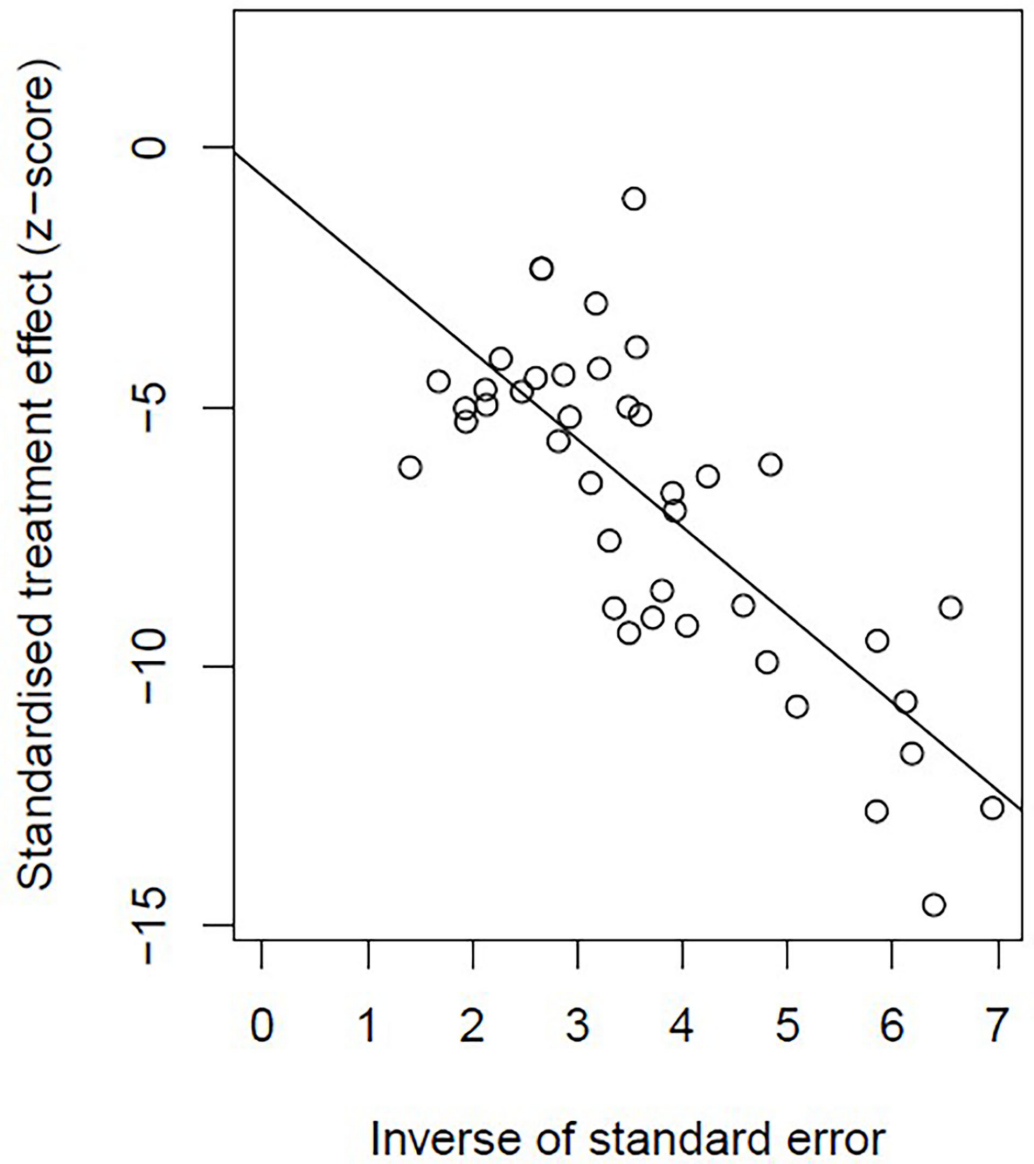
Plot of Egger’s test illustrating the publication bias of meta-analysis of overall pooled incidence rate of hyperprogressive disease.

**Figure 6:**
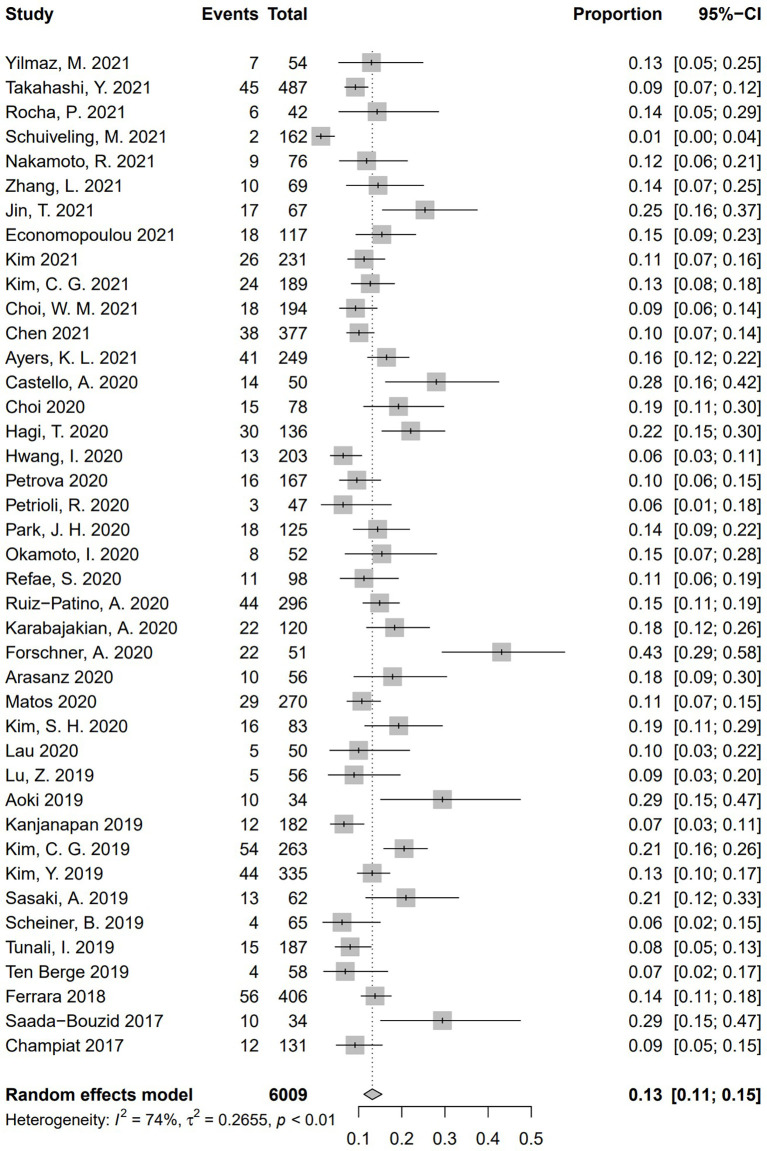
Sensitivity analysis of pooled incidence of hyperprogressive disease in the remaining 40 studies *via* “leave-one-out” method. The “Study” column refers to the study omitted in each time of analysis. CI, confidence interval.

**Figure 7:**
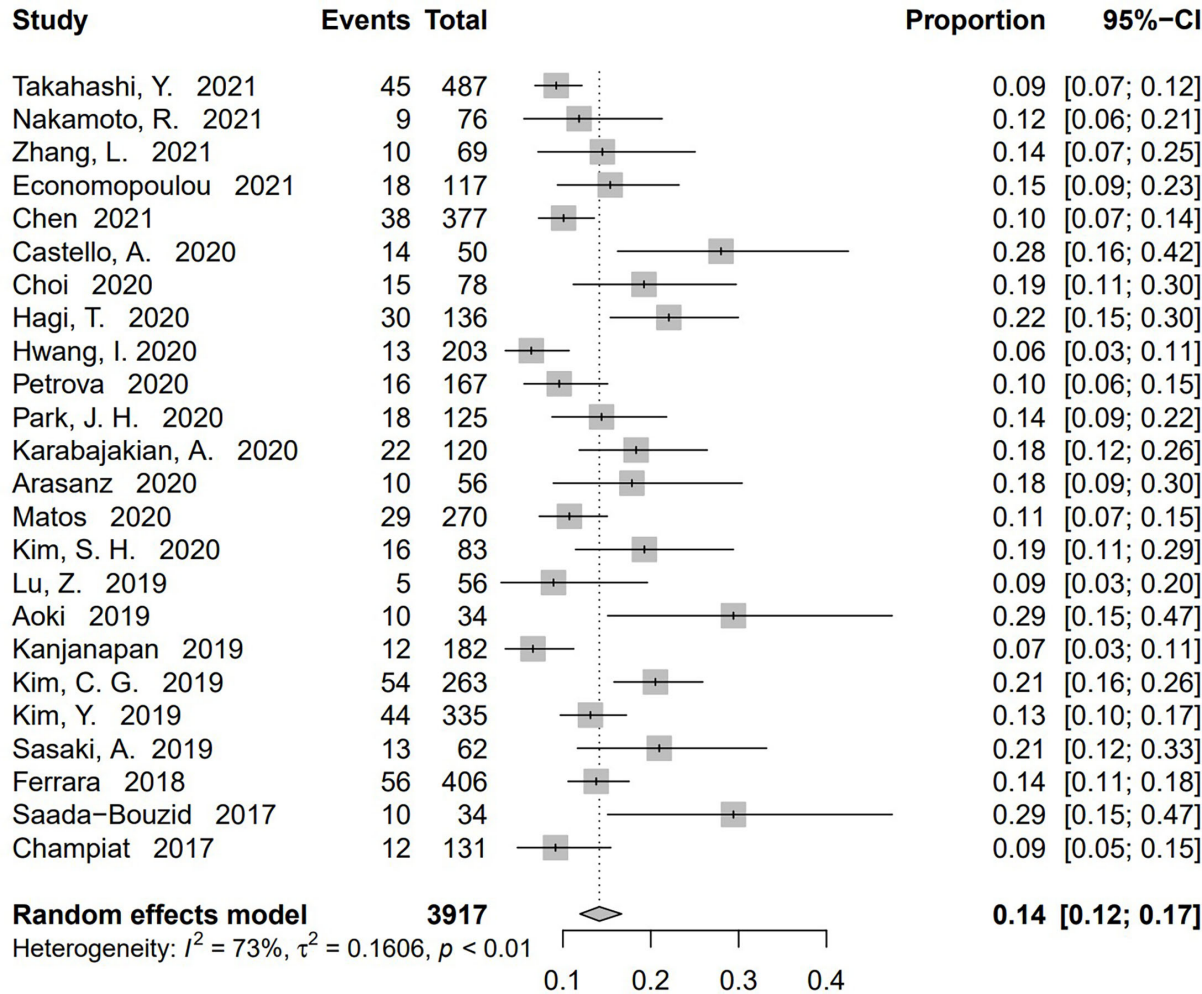
Sensitivity analysis of pooled incidence of hyperprogressive disease in the studies with Newcastle–Ottawa scale (NOS) ≥ 7 *via* “leave-one-out” method. The “Study” column refers to the study omitted in each time of analysis. CI, confidence interval.

Incidence of HPD varied from 1.2% to 43.1%. Because a significant heterogeneity was found (I^2^ = 74%, *P* <.01), the pooled incidence of HPD was 13.2% [95% confidence interval (CI), 11.2%–15.4%] through a random effects model **(** **Figure 3** **)**. In studies led by Matos and Kim providing two approaches to HPD definition, the definition of HPD was chosen based on RECIST 1.1 **(** **Table 1** **) (** 20, 59). No publication bias was found based on either a symmetry funnel plot by visual inspection (**Figure 4**) or quantitative analysis *via* Egger’s test (*P* = .54, **Figure 5**). Regarding sensitivity analysis, we used two approaches to perform it. For the first one, we conducted the analysis for all 41 studies. Using the “leave-one-out” method, the range of pooled incidence of HPD in the remaining 40 included studies was from 12.8% to 13.7% **(** **Figure 6** **)**; for the other one, we specifically included studies with an NOS score not less than seven. Twenty-four studies met the requirement, and the pooled incidence of HPD was 14.1% (95% CI, 12.0%–16.7%). Through sensitivity analysis, the pooled incidence of HPD was between 13.8% and 14.6% **(** **Figure 7** **)**. Whichever the approach, both of the sensitivity analyses illustrated the robustness of the pooled incidence of HPD.

**Figure 8:**
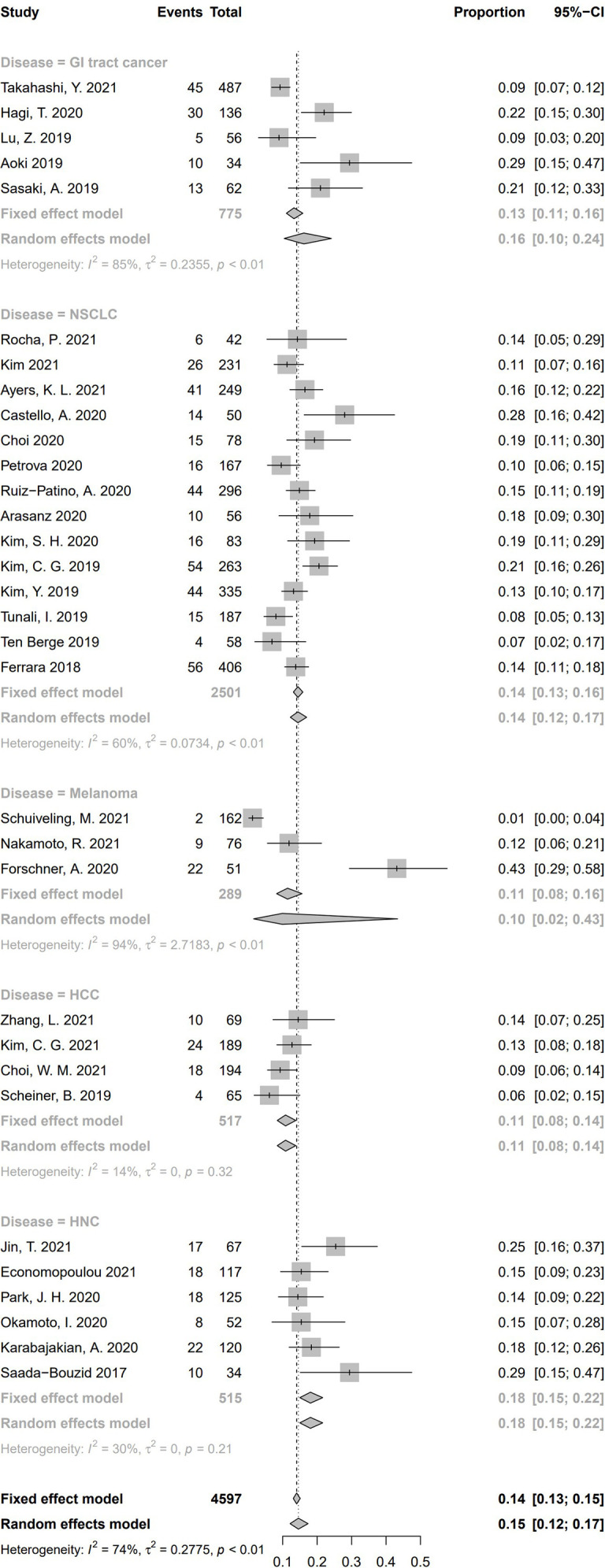
Subgroup analysis regarding types of tumors and incidence of hyperprogressive disease. CI, confidence interval; GI tract, Gastrointestinal tract; HCC, hepatocellular carcinoma; HNC, head and neck cancers; NSCLC, Non-small cell lung cancer.

To conduct the subgroup analysis of incidence of HPD according to tumor type, we selected 32 studies, including only one kind of disease. Illustrated by **Figure 8**, a significant discrepancy was found among the five subgroups (*P* = .02). The pooled incidence of HPD was 16.2% in patients suffering from gastrointestinal (GI) tract cancer (*n* = 5, 95% CI, 10.4%–24.1%) (33, 45, 56, 57, 60); 14.4% in patients with NSCLC (*n* = 14, 95% CI, 12.2%–17.0%) (13, 16, 17, 21, 29, 34, 42–44, 51, 54, 59, 62, 63), 9.9% in patients with melanoma (*n* = 3, 95% CI, 1.6%–43.4%) (35, 36, 53); 10.8% in patients with HCC (*n* = 4, 95% CI, 8.4%–13.8%) (14, 39, 40, 61); 18.06% in patients with HNC (*n* = 6, 95% CI, 15.0%–21.6%) (15, 37, 38, 48, 49, 52). Significant differences were found between these five tumor types (Q = 11.16, *P* = .0248), indicating that tumor type is a source of heterogeneity. Nine studies enrolled several types of cancers and could not be included in tumor type–based subgroup analysis (5, 20, 32, 41, 46, 47, 50, 55, 58).

**Figure 9:**
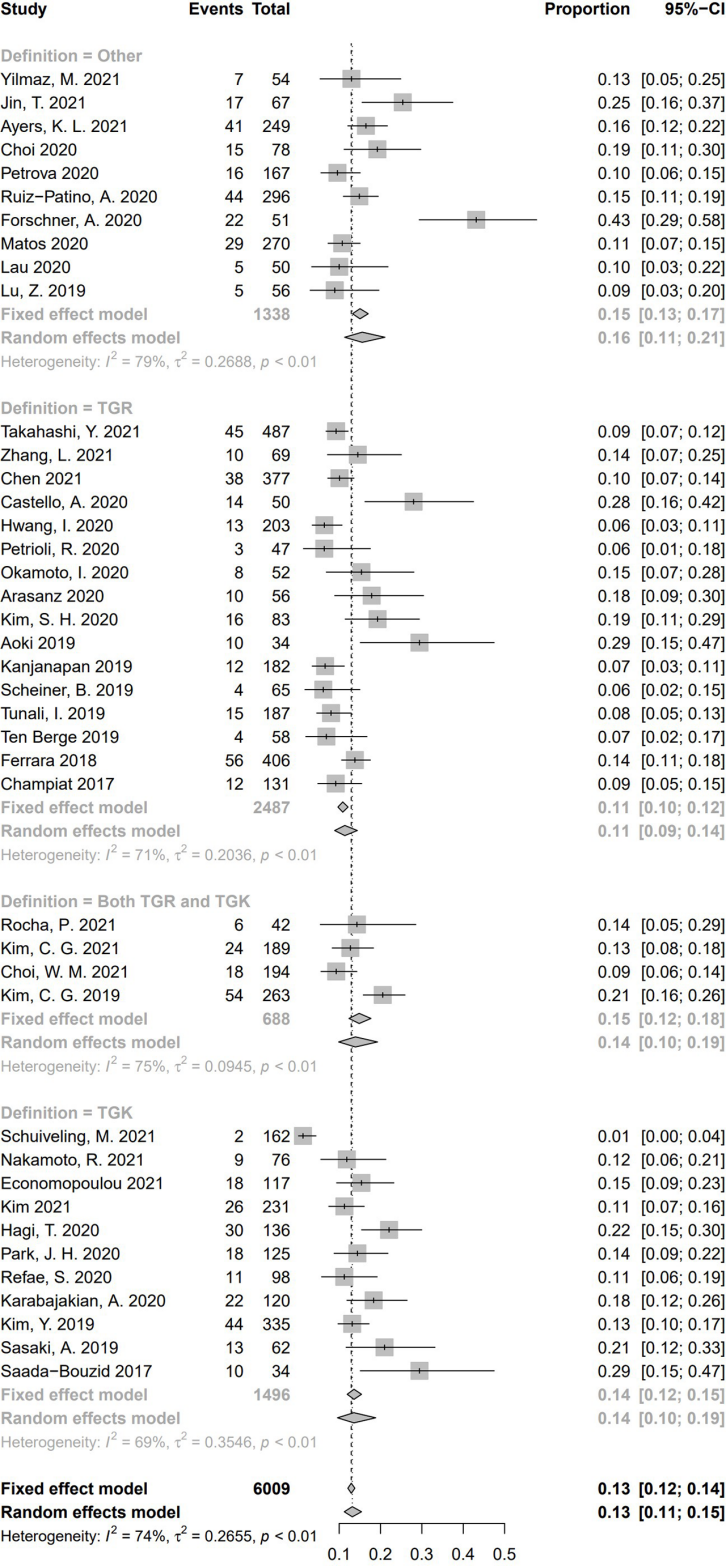
Subgroup analysis regarding hyperprogressive disease’s definitions and its incidence. CI, confidence interval.

According to the definition of HPD, 16 studies calculated TGR only, 11 studies calculated TGK only, four studies included both TGR and TGK in the criteria of HPD, and 10 studies defined HPD by other criteria. Pooled incidence of HPD in the subgroups according to the category of definition of HPD is shown in **Figure 9**. Among the pooled incidence of HPD in four categories, studies using other definitions got the highest pooled incidence (15.6%, 95% CI, 11.3%–21.1%), whereas studies defining HPD with TGR resulted in the lowest pooled incidence (11.4%, 95% CI, 8.9%–14.4%). Studies whose criteria were based on TGK was 13.6% (95% CI, 9.6%–18.8%), and studies using both TGK and TGR was 13.9% (95% CI, 9.9%–19.3%). No significant difference of HPD incidence was found between these four subgroups (Q = 2.68, *P* = .44), which indicates that HPD definition is not a source of heterogeneity.

**Figure 10:**
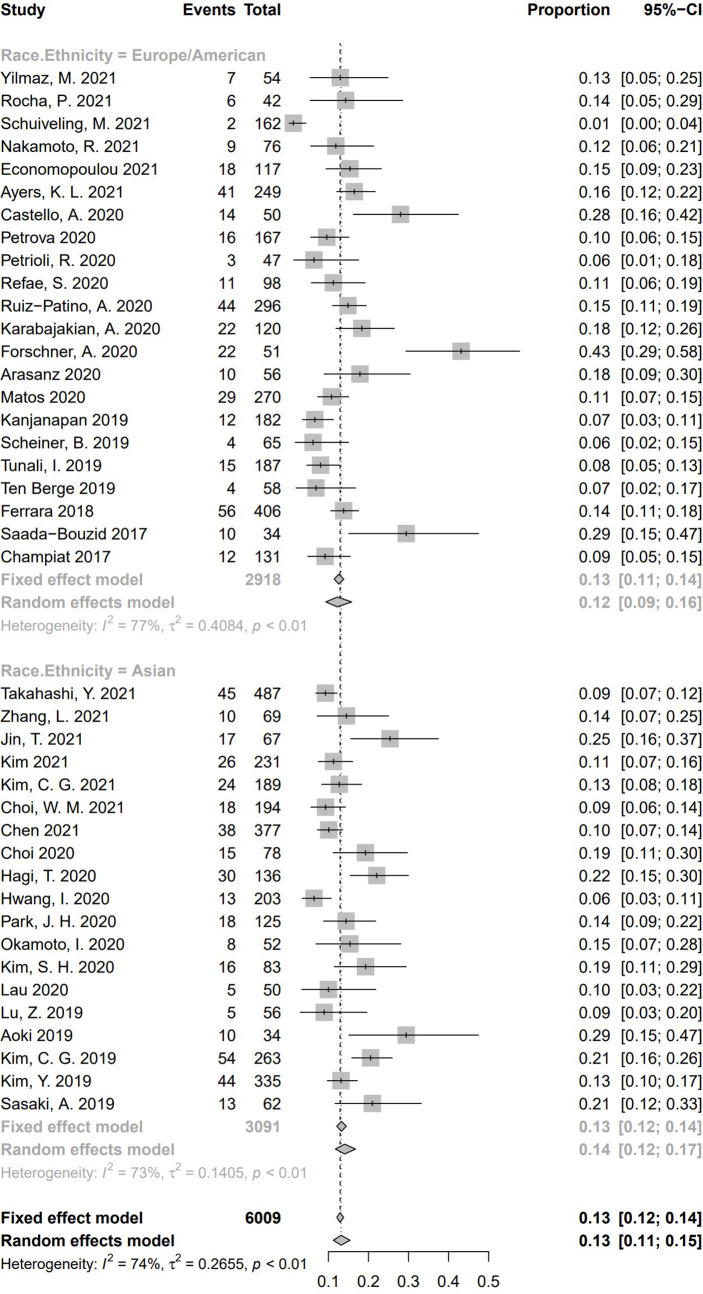
Subgroup analysis regarding race/ethnicity and incidence of hyperprogressive disease. CI, confidence interval.

In all these 41 studies, 19 medical centers were from Asia, and the other 22 centers were European or American. For the subgroup analysis based on race/ethnicity **(** **Figure 10** **)**, the pooled incidence of HPD was 14.1% (95% CI, 11.7%–16.8%) in the 19 Asian studies, whereas a pooled incidence of 12.2% (95% CI, 9.3%–15.8%) was in 22 studies from Europe/America. No significant difference of HPD incidence was demonstrated between the two subgroups (Q = 0.75, *P* = .39), indicating race/ethnicity is not a source of heterogeneity for pooled incidence of HPD.

**Figure 11:**
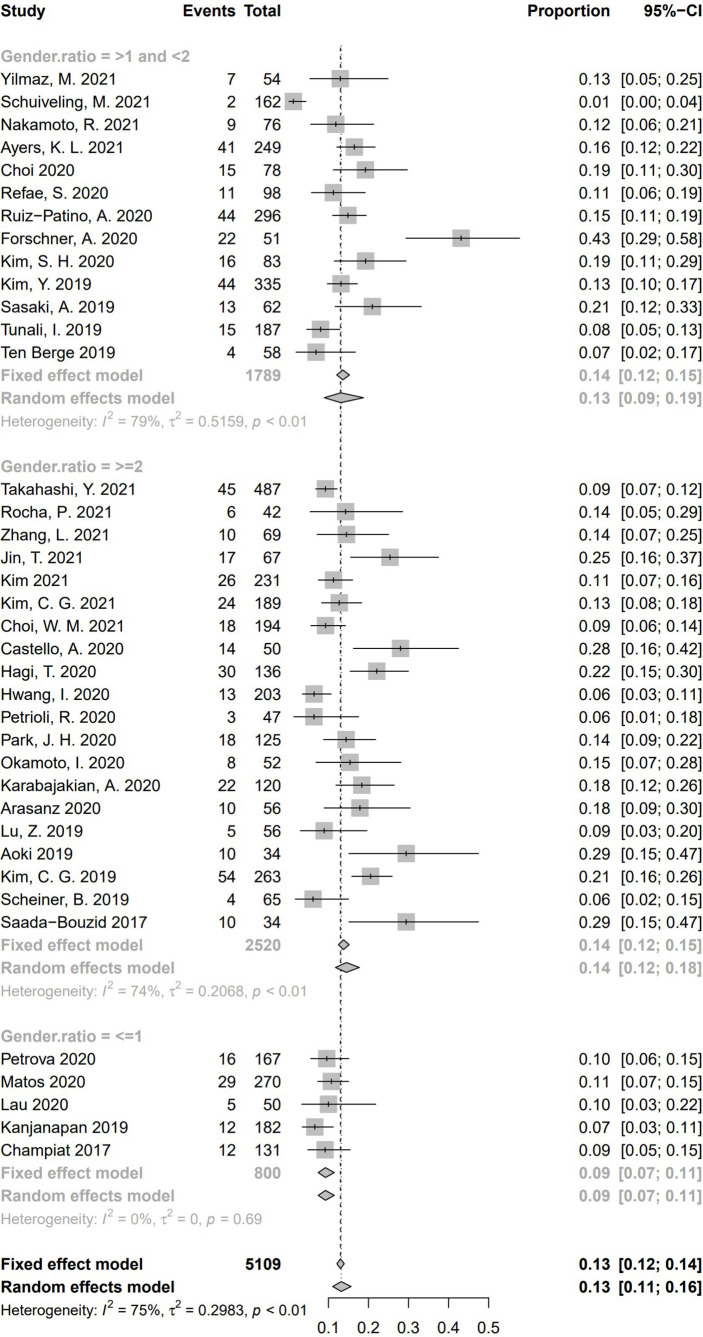
Subgroup analysis regarding gender ratio (male:female) and incidence of hyperprogressive disease. CI, confidence interval.

To explore whether different gender ratio contributed to heterogeneity of HPD, we collected 38 studies having reported numbers of male and female subjects. We separated these studies according to the ratio between male and female [male:female ≥ 2 (*n* = 20), more than one and less than two (*n* = 13), ≤ 1 (*n* = 5)]. Pooled incidence rate of studies with gender ratio ≥ 2 was 14.5% (95% CI, 11.7%–17.7%); 13.1% of studies with a gender ratio between one and two (95% CI, 8.9%–18.8%), and 9.25% in studies with gender ratio no more than one (95% CI, 7.4%–11.5%). Significant difference of HPD incidence was found among these three subgroups (Q = 8.91, *P* = .01), suggesting a cause of heterogeneity **(** **Figure 11** **)**.

According to the clinicopathological risk factors summarized in **Table 2**, five factors, including serum lactate dehydrogenase (LDH), number of metastatic sites, ECOG score, liver metastasis, and PD-L1 status of tumor prior to immunotherapy were selected for further meta-analysis to explore their potential relationship with incidence of HPD. No significant heterogeneity was found in meta-analysis of these five factors (LDH: I^2^ = 21%, *P* = .25; metastatic site: I^2^ = 11%, *P* = .34; ECOG: I^2^ = 0%, *P* = .68; liver metastasis, I^2^ = 27%, *P* = .20; PD-L1: I^2^ = 0%, *P* = .89). Hence, a fixed effect model was used for elaboration of the following results. All the five factors showed significant association with the odds of HPD. Among them, only a positive PD-L1 in the tumor cell served as a protective element for occurrence of HPD (OR = 0.61, 95% CI = 0.42–0.90, *P* = .01), whereas the other four factors (an abnormally high LDH level, number of metastatic sites > 2, ECOG score ≥ 2 and liver metastasis) were all associated with a higher incidence rate of HPD (LDH: OR = 1.51, 95% CI= 1.11–2.06, *P* = .01; metastatic site: OR = 2.38, 95% CI = 1.79–3.18, *P* <.0001; ECOG score: OR = 1.47, 95% CI = 1.06–2.04, *P* = .02; liver metastasis: OR = 3.06, 95% CI = 2.21–4.25, *P* <.0001). Forest plots of these five predictors of HPD are demonstrated in **Supplementary Figures 1A–E**. No publication bias was found according to funnel plots and Egger’s tests (for Egger’s test, LDH: *P* = .80; metastatic sites: *P* = .23; ECOG score: *P* = .74; liver metastasis: *P* = .24; PD-L1: *P* = .29) illustrated in **Supplementary Figure 2A–E**, **3A–E**, respectively. In addition to the above risk factors, some studies also found that elevated neutrophil-to-lymphocyte ratio, portal vein thrombosis, and some other high-risk genes (MDM2/4, KRAS, VEGFR, etc.) were also risk factors for HPD though the number of studies supporting these findings were relatively small.

## Discussion

Throughout our analysis, a majority of the included 41 studies had a high quality based on the evaluation of the NOS scale. Total sample size (more than 6000) was large in 41 eligible studies, and each study collected more than 30 cases. Solid malignancies analyzed in eligible studies included NSCLC, HNC, HCC, GI tract cancer, etc. Nivolumab, Pembrolizumab, Camrelizumab, Avelumab, Atezolizumab, Durvalumab, Ipilimumab, and Tremelimumab were relatively popular agents. Incidence of HPD was calculated in all 41 studies. Various criteria of HPD definitions were used in different studies based on the prerequisite of progression of disease according to RECIST 1.1. Main categories include calculation of TGK, TGR, TTF, and other relatively uncommon items, including the increment of total tumor burdens. The pooled incidence of HPD in 41 studies was 13.2% with a range from 1% to 43%. No publication bias was noted, and the result of pooled incidence of HPD was robust.

Regarding clinical outcomes, both the reported median OS and PFS were shorter than two years. Unlike the cohort of non-HPD or non-HPD PD patients, median OS of HPD patients was less than one year. A large number of the included studies found that the prognosis of HPD patients were poorer than that of the non-HPD and natural PD cohorts, no matter for median OS or PFS.

For subgroup analysis, it was found that different tumor types and different gender ratios resulted in different incidence of HPD during ICI treatment, and both are two sources of heterogeneity created by pooled analysis of HPD incidence. Regarding tumor types, the highest pooled incidence of HPD was in patients suffered from HNC (18.06%), followed by GI tract cancer (16.2%) and NSCLC (14.4%). As for gender ratio, a higher proportion of male patients was associated with a higher incidence rate of HPD (14.5%). Of note, the number of studies in the subgroup of gender ratio ≤ 1 was much smaller than the other two subgroups. Hence, whether a higher female proportion was associated with a lower HPD occurrence should be further investigated. In contrast to the results above, the incidence of HPD was not related to types of HPD definition or race/ethnicity according to the subgroup analysis (*P* = .44 and *P* = .39, respectively). For associations of HPD with five potential clinicopathological factors, a positive immunohistochemistry result of PD-L1 of the tumor cell was related to a low risk of HPD, whereas the other four factors (a high LDH level, more than two metastatic sites, liver metastasis, and ECOG ≥ 2) were all predictive factors indicating a higher risk of HPD.

Immunotherapy has shown its effectiveness in various hematological and metastatic/refractory solid malignancies. Previous research shows that approximately 15% to 25% of patients of various types of cancers sensitive to PD-1/PD-L1 and CTLA-4 receptor ICIs (65). Nevertheless, some patients did not response well and even suffered from a greater disease progression during or shortly after the immunotherapy, which is termed HPD. The main clinical features of HPD were rapid tumor enlargement, existence of new lesions in primary or distal tissue and organs, and deterioration of the general condition of patients. Before the confirmation of HPD, a differential diagnosis of pseudo-progression is necessary. In contrast to HPD, pseudo-progression is a temporary increase of tumor size or existence of new lesions but without clinical worsening, followed by self-limited and stable antitumor response (66). For the time of onset of pseudo-progression, it can occur within or after the first 12 weeks of antitumor treatment. At present, this manifestation can be explained as immune cells infiltrating into the tumor or edema/tumor necrosis caused by immunotherapy (66). Methods to distinguish between HPD and pseudo-progression include medical imaging, such as PET/CT; molecular tracers specifically targeting receptors expressed on immunocytes (PD-1, PD-L1, CTLA-4); and liquid biopsy to track chromosomal instability and detect circulating tumor DNA (67–70).

For HPD identification, we found that calculation of TGR is still a popular method for HPD determination. TGK is also a common approach for HPD evaluation. To further reassure clinicians of the HPD diagnosis, some studies calculate both TGR and TGK to verify the final evaluation (29, 34, 39, 40). Other criteria are also used to describe the HPD phenomenon, including “more than 40% or 50% increment of total tumor burden,” “ECOG PS exceeding 2 during anti-tumor treatment,” and “more than two new lesions exist in previously involved organ or new organs.” However, these criteria are not in consensus for the oncological community. Note that 12 studies add “TTF less than 2 months” into the criteria, but other studies do not take TTF as a requirement of HPD diagnosis because evaluation of tumor response was after two months. Cutoff of the time for evaluation of tumor response to justify whether HPD is determined or not is still in hot debate. Nevertheless, our finding suggests that the types of HPD definition do not affect HPD incidence. Different ways of defining HPD are dependent on the actual condition of the data. If medical imaging before immunotherapy is not available, TGR and TGK cannot be figured out. Hence, the tumor burden of target lesions are evaluated by measuring the sum of every unidimensional longest diameter of each lesion. For diffused lesions in some patients, HPD can be identified by finding newly appearing lesions in organs involved or uninvolved previously. Hence, the most proper method can be selected to recognize HPD according to which kind of data are available.

Interestingly, HPD is a special complication related but not restricted to ICIs. In one study published in 2016, some melanoma patients suffered from disease relapse or even death within a short time during the treatment of adjuvant, multi-subtype interferon-α (71). In addition to immunotherapy, several studies found that HPD could also occur in chemotherapy or even radiotherapy. In the Saâda-Bouzid study, nine out of 10 patients with HPD had at least one previous loco-regional recurrence in an irradiated field (15). In a case report from China, a 42-year-old female was diagnosed as having stage IV renal clear cell carcinoma. After a first- and second-line therapy of sorafenib and pazopanib without effectiveness, she accepted a combination therapy of nivolumab (180 mg, 3 mg/kg, every two weeks) as well as radiotherapy six days after the first dose of PD-1 immunotherapy (stereotactic body radiation therapy followed by the conventional irradiation to the right kidney lesion). Eighteen days after the third dose of nivolumab, the patient suffered from a rapid disease progression with a deteriorating general condition and a rapid increase of metastatic lesions in her left lung. Biopsy of the lung focus confirmed the HPD as no lymphocyte infiltration was detected, excluding the possibility of pseudo-progression. Despite salvage treatment *via* prednisone therapy, the patient unfortunately died 70 days after the third cycle of nivolumab (28). Another two case reports also supported the relationship between radiotherapy followed by immunotherapy and the occurrence of HPD (72, 73). The potential mechanism of this phenomenon was that radiotherapy can be harmful to the immune system. Researchers propose that previous radiotherapy increases the secretion of type-I interferon by tumor cells or immune cells and suppress the tumor-infiltrating lymphocytes (72). Moreover, Schaue et al. suggests that radiotherapy can elevate the relative abundance of regulator T cells, which need to be justified by further investigations (74). Still, no clinical study explored the relationship between HPD and pure radiation therapy. Chemotherapy is another cause of HPD as well. In a phase-III clinical trial of atezolizumab vs. docetaxel for NSCLC, incidence of HPD was similar between the atezolizumab and docetaxel groups (10.4% vs. 9.6%, respectively). This result indicates that HPD is not specific to immunotherapy (75). In the Jin study, however, the cohort of NPC patients received a combination of anti-PD1 ICI, and chemotherapy had a much lower rate of HPD than those that received only anti-PD1 ICI (39% vs. 3.8%). The great discrepancy of HPD incidence between the two groups may be because of the prevention against disease progression by chemotherapy (37). Likewise, in a study comparing HPD rates between NSCLC patients treated with ICI monotherapy and patients treated with ICI and chemotherapy, incidence of HPD in the former group was significantly higher than in the latter group (17.6% vs. 2.9%, *P* = .031). In a Japanese study investigating incidence of HPD in patients suffering from gastric cancer who received nivolumab or irinotecan, no significant difference of HPD rates between these two groups (28.1% vs. 13.5%, *P* = .08) (76). Therefore, whether chemotherapy is a preventive or a stimulative factor for the occurrence of HPD warrants further study.

We summarize the major risk factors for HPD in 41 included studies in **Table 2**. Common risk factors include age, tumor size, metastatic burden, ECOG score, elevation of neutrophil-lymphocyte ratio, and/or LDH and genomic alterations (MDM2/4 amplification, EGFR aberration, VEGFR2 variation). For the relationship between HPD and age, whether a younger or older age related to HPD was not decided. The Economopoulou, Park, and Choi studies suggest younger age is a risk factor for HPD (38, 44, 48), whereas the studies published by Champiat, Hwang, and Refae point to elderly patients being disposed to suffer from HPD during or after immunotherapy (5, 46, 50). A potential mechanism is related to the ineffectiveness of intracellular antigens and the immune microenvironment in which immune cells and chemokines play a key role (77). As for the relationship between HPD and gender, the study by Kanjanapan was temporarily the only study reporting that women had a higher risk of HPD when receiving immunotherapy (58). However, this conclusion is not in line with the result in our study, in which a higher sample size ratio of male to female is associated with a higher rate of HPD. Six of 41 studies support the association of high neutrophil-lymphocyte ratio (NLR) with occurrence of HPD. For the Kim study, a high NLR at baseline (NLR > 6) was the only risk factor for HPD. They also found that a cutoff value of 4.125 is optimal to predict HPD (39). NLR is equal to the ratio of peripheral neutrophil count divided by peripheral count of lymphocytes. Peripheral neutrophil includes tumor-associated neutrophil (TAN) and myeloid-derived suppressor cells (MDSCs). TANs recruit regulatory T cells and MDSC *via* the production of chemokines, causing tumor progression (78). As a result, the numerator of the NLR represents the immunosuppressive element, and its increment is counteractive against antitumor activity (40). The denominator of NLR stands for antitumor cytotoxic T cells and a decrease of this value means a weakened antitumor immune response by effector T lymphocytes (79). Hence, the change of NLR reflects the change of the tumor microenvironment during antitumor immunotherapy. Gene alteration is an essential trait not only for common cancers, but also in malignancies with the potential of occurring HPD. Somatic alteration of MDM2/4, EGFR, and other genes occurred on chromosome 11q13 (80). As a proto-oncogene encoding E3 ubiquitin ligase, MDM2 promoted degradation of p53, an important tumor suppressor, resulting in carcinogenesis (81, 82). A previous study suggests that amplification of MDM2 was associated with rapid tumor growth in patients receiving immunotherapy (18). A study led by Zou et al. found that MDM2 could also inhibit T cell activation *via* degrading transcription factor NFATc2, leading to the resistance of anti-PD-1 medications (83). As a tumor-associated antigen, MDM2 overexpression could also induce immunologic tolerance in HPD (84, 85). Still, other roles and further mechanisms of MDM2, especially interactions with other molecules, need to be investigated.

Some other clinicopathological factors also played an important role in HPD prediction. As a common serum biomarker, LDH at first was used as a diagnostic molecule for liver dysfunction, myocardial infarction, and myopathies (86–88). Besides this, some studies found that LDH is also an indicator in cancer patients. Its elevation is related to tumor recurrence and metastasis, which is proved in several solid malignancies (89, 90). Our study further proves the contribution of LDH in finding that an abnormally high LDH is associated with a higher risk of HPD, which led to a poor prognosis. PD-1 is a common immune checkpoint molecule expressed by activated T cells, whereas PD-L1 is a famous biomarker mainly expressed on cancer cells and antigen-presenting cells. As immunosuppressive PD-1 protein binds to PD-L1, T cell activation is suppressed, and immune evasion of tumor cells is stimulated (91). Anti-PD-1 or anti-PD-L1 monoclonal antibodies are capable of ending immunosuppression and resuming antitumor T cell response by blocking the binding of PD-1 on T cells and PD-L1 on tumor cells (92). Therefore, a positive PD-L1 expression on tumor cells provides targets for ICIs to inhibit T cell immunosuppression, hindering tumor growth, reducing tumor burden, and lowering the risk of HPD during immunotherapy. Personally, blood LDH and tumor PD-L1 expression serve as “barometers” to predict HPD occurrence for solid tumor patients receiving immunotherapy.

Our study has some limitations to be considered. 1) The majority of eligible studies were retrospective research, so validation of the feasibility and accuracy of HPD criteria (TGR, TGK, TTF, and other items) could not be conducted in published clinical studies in that the data of medical imaging in three time points (prebaseline, baseline, and posttreatment) could not be retrieved; 2) because of the heterogeneous result of survival data, meta-analysis of OS and PFS of HPD failed to perform.

## Conclusions

The pooled incidence of HPD was 13.2% in all 41 included studies. The definition of HPD was classified to four main categories, but did not affect the ultimate incidence of HPD among different studies. Serum LDH and tumor immunohistochemistry prior to immunotherapy (PD-L1) will play an important role in predicting the occurrence of HPD in future clinical practice.

## Data availability statement

The original contributions presented in the study are included in the article/**Supplementary Material**. Further inquiries can be directed to the corresponding author.

## Author contributions

ZJZ and XL conceived and designed the research. ZJZ prepared the manuscript. ZJZ, JB, JWZ and TZ collected the data. ZJZ and JB analyzed the data. XL made the final revisions. All authors contributed to the article and approved the submitted version.

## Funding

This work was supported by CAMS Innovation Fund for Medical Sciences (CIFMS) (2021-I2M-1-061 and 2021-1-I2M-003 and 2018-I2M-3-001); CAMS Clinical and Translational Medicine Research Funds (2019XK320006), Beijing Natural Science Foundation (7192158), CSCO-hengrui Cancer Research Fund (Y-HR2019-0239) and National Ten-thousand Talent Program.

## Acknowledgments

We deeply appreciated Prof. Changtai Zhu from Shanghai Jiaotong University for pertinent advice and suggestion of data extraction, summary and analysis.

## Conflict of interest

The authors declare that the research was conducted in the absence of any commercial or financial relationships that could be construed as a potential conflict of interest.

## Publisher’s note

All claims expressed in this article are solely those of the authors and do not necessarily represent those of their affiliated organizations, or those of the publisher, the editors and the reviewers. Any product that may be evaluated in this article, or claim that may be made by its manufacturer, is not guaranteed or endorsed by the publisher.

## Abbreviations

CI, Confidence interval; CTLA-4, Cytotoxic T lymphocyte-associated protein 4; ECOG PS, Eastern Cooperative Oncology Group Performance Score; GI tract, Gastrointestinal tract; HCC, Hepatocellular carcinoma; HNC, Head and neck cancers; HNSCC, Head and neck squamous cell carcinoma; HPD, Hyperprogressive disease; ICI, Immune checkpoint inhibitors; iRECIST, Immune RECIST; irRC, Immune-related response criteria; irRECIST, Immune-related RECIST; LDH, Lactate dehydrogenase; NOS, Newcastle-Ottawa Scale; NSCLC, Non-small cell lung cancer; OR, Odds ratio; OS, Overall survival; PD, Progressive disease; PD-1, Programmed cell death protein 1; PD-L1, Programmed cell death protein 1 ligand 1; PFS, Progression-free survival; RECIST, Response evaluation criteria in Solid Tumors; TGK, Tumor growth kinetics; TGR, Tumor growth rate.
