# Supplementary material for: Hyperprogressive disease in patients suffering from solid malignancies treated by immune checkpoint inhibitors: A systematic review and meta-analysis
Source: Front Oncol. 2022 Aug 3;12:843707. doi: 10.3389/fonc.2022.843707 (PMC9381837; doi:10.3389/fonc.2022.843707)
Supplement: Supplementary file 1 [file DataSheet_1.docx]

**Supplementary Table 1. Search strategy in database of Pubmed, Embase and Cochrane library**

| **Pubmed (N=303)** |
| --- |
| (("hyperprogressive*"[Title/Abstract]) OR ("hyperprogression"[Title/Abstract])) OR ("hyperprogressor*"[Title/Abstract]) |
| **Embase (N=469)** |
| ('hyperprogressive disease'/exp) OR (hyperprogressive*:ab,ti OR hyperprogression:ab,ti OR hyperprogressor*:ab,ti) |
| **Cochrane library (N=18)** |
| [(hyperprogressive*):ti,ab,kw] OR [(hyperprogressor*):ti,ab,kw] OR [(hyperprogression):ti,ab,kw] |

**Supplementary Table 2. The Newcastle-Ottawa Scale Quality Assessment of the eligible studies**

| **Study** | **Selection of cohorts** | | | | **COMPARABILITY** | **OUTCOME** | | | **NOS**  **Score** |
| --- | --- | --- | --- | --- | --- | --- | --- | --- | --- |
|  | Representativeness of the Exposed Cohort^1^ | Selection of the Non-Exposed Cohort | Ascertainment of Exposure | Demonstration That Outcome of Interest Was Not Present at Start of Study^2^ | Comparability of Cohorts on the Basis of the Design or Analysis^3^ | Assessment of Outcome | Was Follow-Up Long Enough for Outcomes to Occur^4^ | Adequacy of Follow Up of Cohorts^5^ |  |
| Yilmaz, M. et.al (2021) | **☆** | **☆** | **☆** |  |  | **☆** |  |  | 4 |
| Takahashi, Y. et.al (2021) | **☆** | **☆** | **☆** | **☆** |  | **☆** | **☆** | **☆** | 7 |
| Rocha, P. et.al (2021) | **☆** | **☆** | **☆** |  |  | **☆** | **☆** | **☆** | 6 |
| Schuiveling,M. et.al (2021) | **☆** | **☆** | **☆** |  | **☆** | **☆** |  |  | 5 |
| Nakamoto, R. et.al (2021) | **☆** | **☆** | **☆** |  | **☆☆** | **☆** | **☆** | **☆** | 8 |
| Lu Zhang et.al (2021) | **☆** | **☆** | **☆** |  | **☆** | **☆** | **☆** | **☆** | 7 |
| Jin, T. et.al (2021) | **☆** | **☆** | **☆** |  | **☆☆** | **☆** |  |  | 6 |
| Economopoulou et.al (2021) | **☆** | **☆** | **☆** |  | **☆☆** | **☆** | **☆** | **☆** | 8 |
| Kim et.al (2021) | **☆** | **☆** | **☆** |  | **☆** | **☆** |  |  | 5 |
| Kim, C. G. et.al (2021) | **☆** | **☆** | **☆** |  |  | **☆** | **☆** | **☆** | 6 |
| Choi, W. M. et.al (2021) | **☆** | **☆** | **☆** |  |  | **☆** |  |  | 4 |
| Chen et.al (2021) | **☆** | **☆** | **☆** |  | **☆☆** | **☆** | **☆** | **☆** | 8 |
| Ayers, K. L. et.al (2021) | **☆** | **☆** | **☆** |  |  | **☆** |  |  | 4 |
| Castello, A. et.al (2020) | **☆** | **☆** | **☆** | **☆** | **☆☆** | **☆** | **☆** | **☆** | 9 |
| Choi et.al (2020) | **☆** | **☆** | **☆** |  | **☆** | **☆** | **☆** | **☆** | 7 |
| Hagi, T. et.al (2020) | **☆** | **☆** | **☆** |  | **☆☆** | **☆** | **☆** | **☆** | 8 |
| Hwang, I. et.al; (2020) | **☆** | **☆** | **☆** |  | **☆** | **☆** | **☆** | **☆** | 7 |
| Petrova et.al (2020) | **☆** | **☆** | **☆** |  | **☆☆** | **☆** | **☆** | **☆** | 8 |
| Petrioli, R. et.al (2020) | **☆** | **☆** | **☆** |  |  | **☆** |  |  | 4 |
| Park, J. H. et.al (2020) | **☆** | **☆** | **☆** |  | **☆** | **☆** | **☆** | **☆** | 7 |
| Okamoto, I. et.al (2020) | **☆** | **☆** | **☆** |  |  | **☆** |  |  | 4 |
| Refae, S. et.al (2020) | **☆** | **☆** | **☆** |  | **☆** | **☆** |  |  | 5 |
| Ruiz-Patiño, A. et.al (2020) | **☆** | **☆** | **☆** |  | **☆** | **☆** |  |  | 5 |
| Karabajakian, A. et.al (2020) | **☆** | **☆** | **☆** |  | **☆☆** | **☆** | **☆** | **☆** | 8 |
| Forschner, A. et.al (2020) | **☆** | **☆** | **☆** |  | **☆☆** | **☆** |  |  | 6 |
| Arasanz et.al (2020) | **☆** | **☆** | **☆** | **☆** | **☆☆** | **☆** | **☆** | **☆** | 9 |
| Matos et.al (2020) | **☆** | **☆** | **☆** | **☆** | **☆☆** | **☆** | **☆** | **☆** | 9 |
| Kim, S. H. et.al (2020) | **☆** | **☆** | **☆** |  | **☆☆** | **☆** | **☆** | **☆** | 8 |
| Lau et.al (2020) | **☆** | **☆** | **☆** |  |  | **☆** |  |  | 4 |
| Lu, Z. et.al (2019) | **☆** | **☆** | **☆** |  | **☆☆** | **☆** | **☆** | **☆** | 8 |
| Aoki et.al (2019) | **☆** | **☆** | **☆** |  | **☆☆** | **☆** | **☆** | **☆** | 8 |
| Kanjanapan et.al (2019) | **☆** | **☆** | **☆** |  | **☆☆** | **☆** | **☆** | **☆** | 8 |
| Kim, C. G. et.al (2019) | **☆** | **☆** | **☆** |  | **☆☆** | **☆** | **☆** | **☆** | 8 |
| Kim, Y. et.al (2019) | **☆** | **☆** | **☆** |  | **☆☆** | **☆** | **☆** | **☆** | 8 |
| Sasaki, A. et.al (2019) | **☆** | **☆** | **☆** |  | **☆☆** | **☆** | **☆** | **☆** | 8 |
| Scheiner, B. et.al (2019) | **☆** | **☆** | **☆** |  |  | **☆** |  |  | 4 |
| Tunali, I. et.al (2019) | **☆** | **☆** | **☆** | **☆** |  | **☆** |  |  | 5 |
| Ten Berge et.al (2019) | **☆** | **☆** | **☆** |  |  | **☆** | **☆** | **☆** | 6 |
| Ferrara et.al (2018) | **☆** | **☆** | **☆** |  | **☆☆** | **☆** | **☆** | **☆** | 8 |
| Saâda-Bouzid et.al (2017) | **☆** | **☆** | **☆** |  | **☆** | **☆** | **☆** | **☆** | 7 |
| Champiat et.al (2017) | **☆** | **☆** | **☆** | **☆** | **☆** | **☆** | **☆** | **☆** | 8 |

Total score was 9 stars for each study. A study can be awarded a maximum of one star for each numbered item within the Selection and Exposure categories. A maximum of two stars can be given for Comparability.

1 Exposure was referred to the occurrence of hyperprogression. Each study was prized one star for the first three items in that hyperprogression cohort were genuine or representative and they were not deliberately recruited from general population with hyperprogressive disease.

2 Only prospective study would be given one star.

3 If a study was adjusted for age, one star was awarded, and if a study adjusted for any additional factors (e.g., gender, tumor size, ECOG status, histological type, number of metastasis site, number of prior chemotherapy lines, types of immunotherapy, any laboratory tests, etc.), an additional star was awarded.

4 One point would be given for studies demonstrating the results of OS or PFS as well as making a comparison between circumstances of hyperprogression and non-hyperprogression or between hyperprogressions and non-hyperprogression disease progression,

5 one star would be provided for studies with a follow-up rate of exceeding 65%.

NOS, Newcastle-Ottawa Scale.


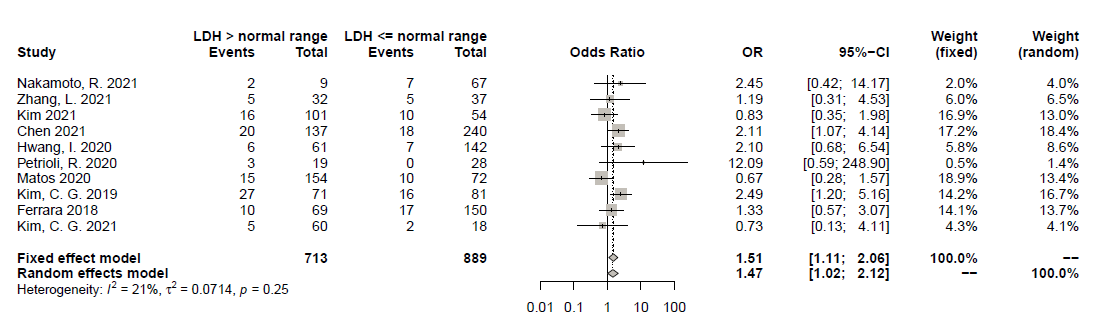


**A**


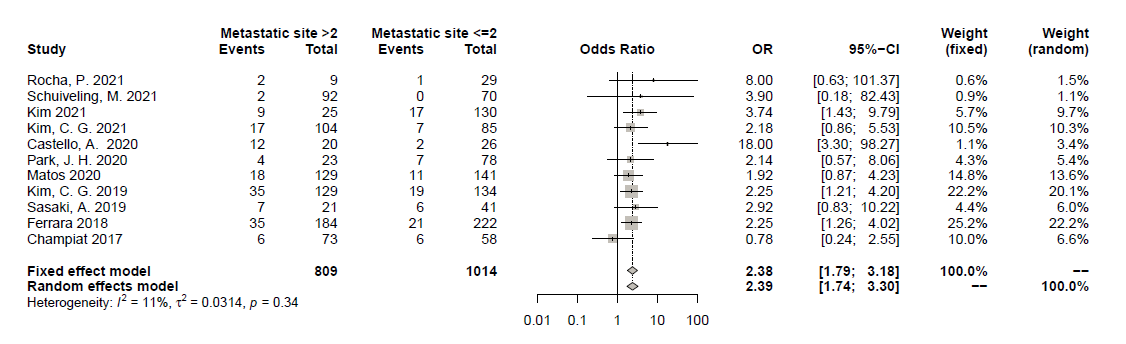


**B**


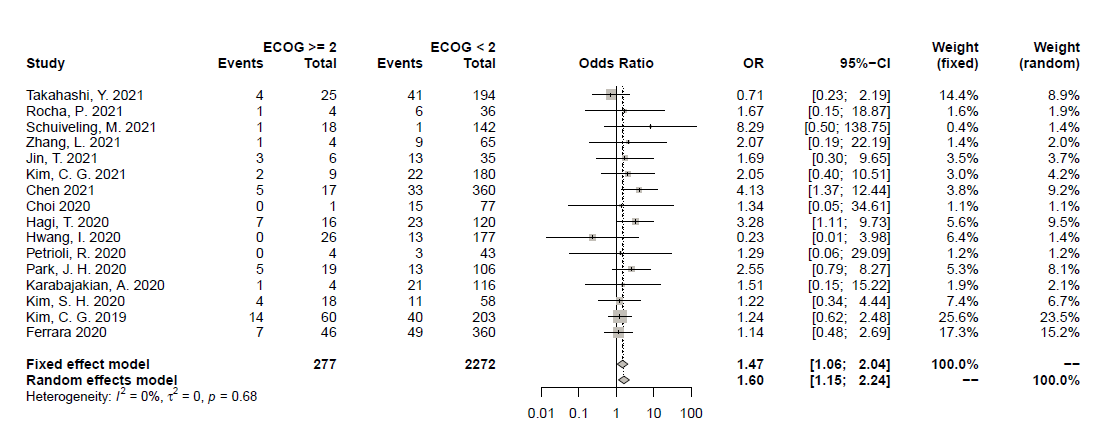


**C**


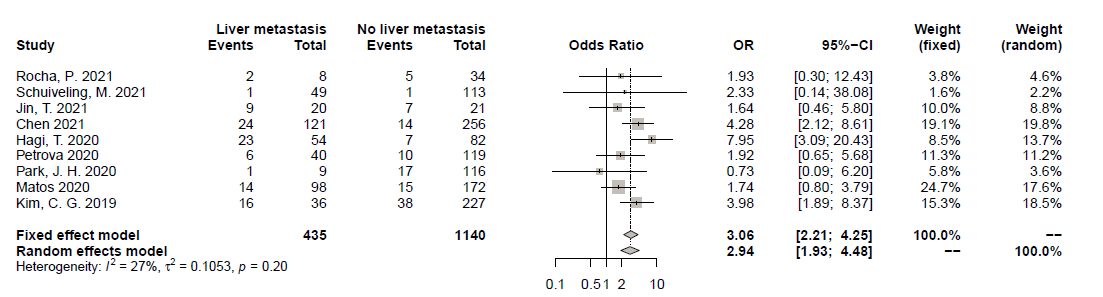


**D**


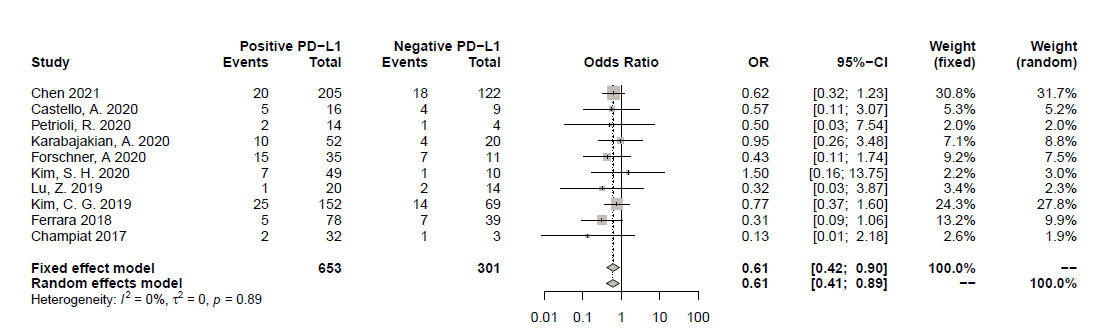


**E**

**Supplementary Figure 1. Forest plots illustrating the associations of potential risk factors at baseline status with hyperprogressive disease events. Serum LDH above/within the normal range (A); More**

**than /no more than two metastatic sites (B); ECOG score ≥2/<2 (C); Liver metastases/No liver metastasis (D); Positivity/Negativity tumor PD-L1 expression status (E).**

CI, confidence interval; ECOG, Eastern Cooperative Oncology Group; LDH, lactate dehydrogenase; OR, odds ratio; PD-L1, programmed death 1 ligand 1.


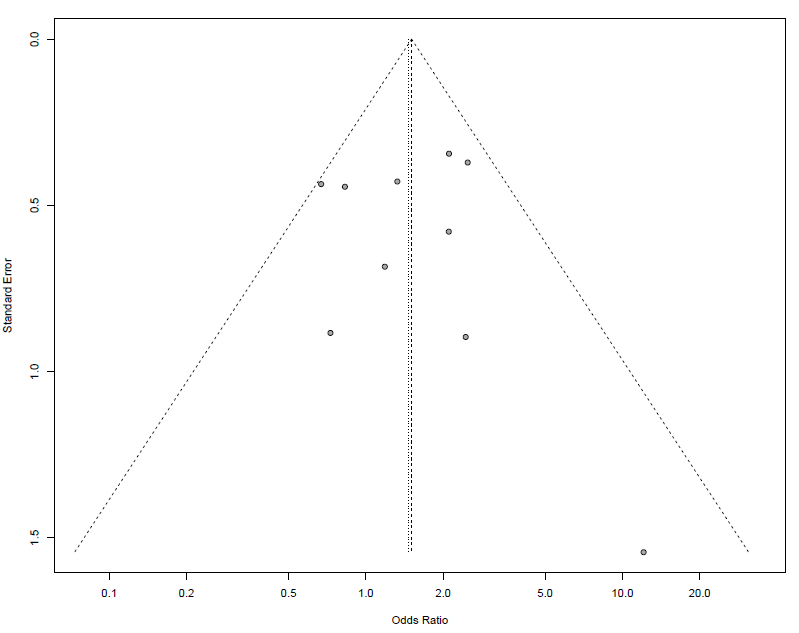


**A**


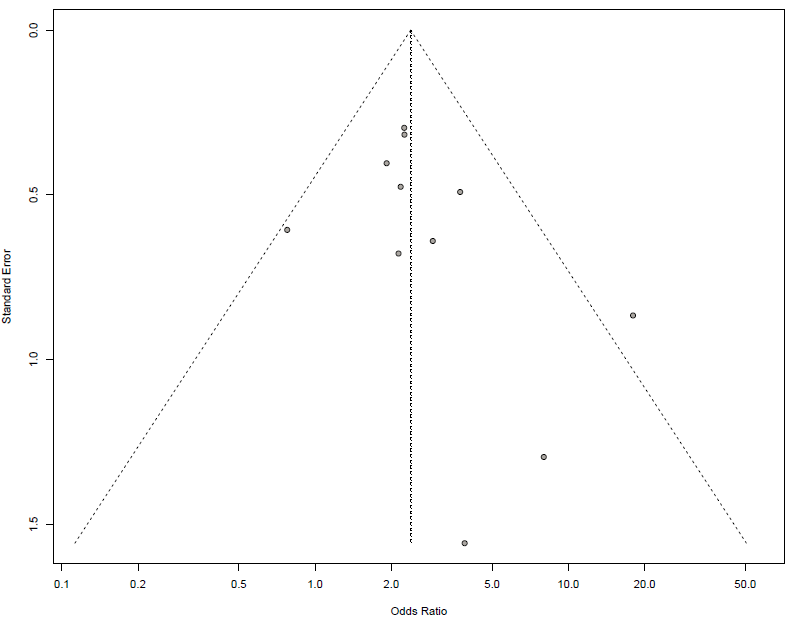


**B**


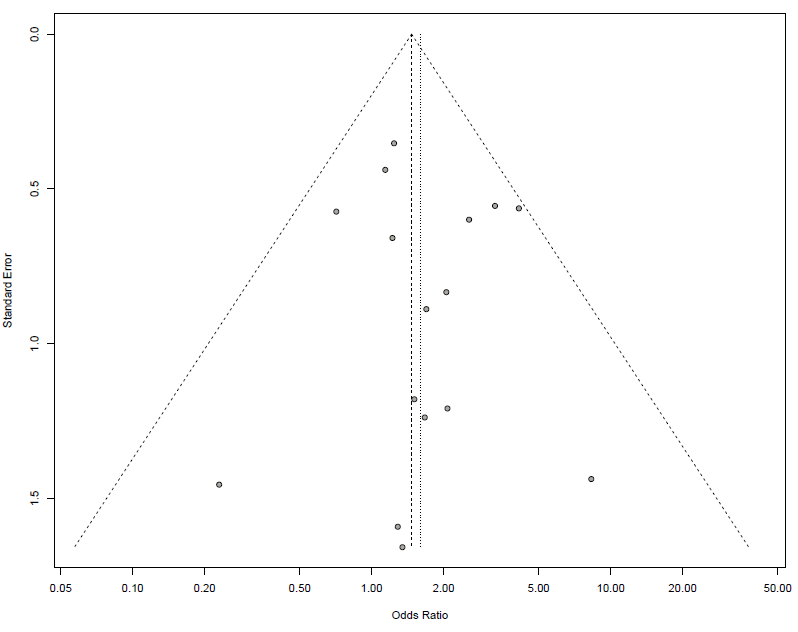


**C**


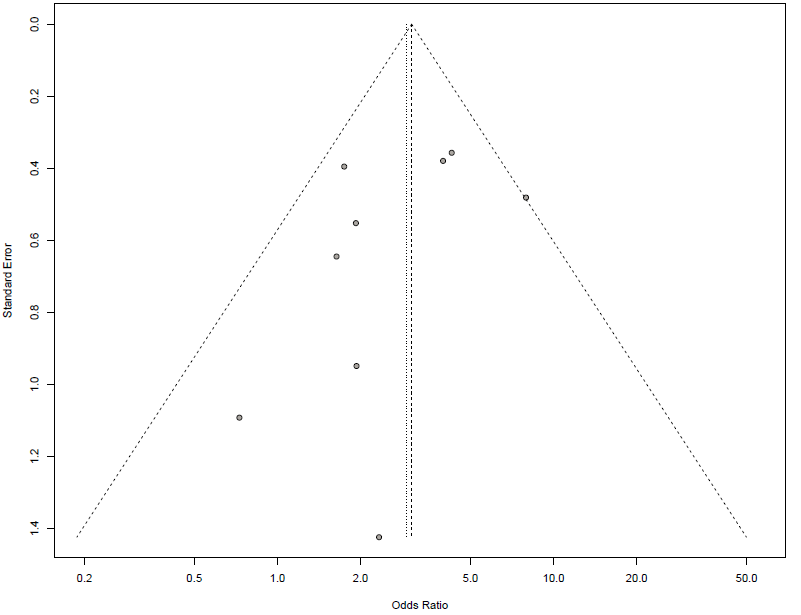


**D**


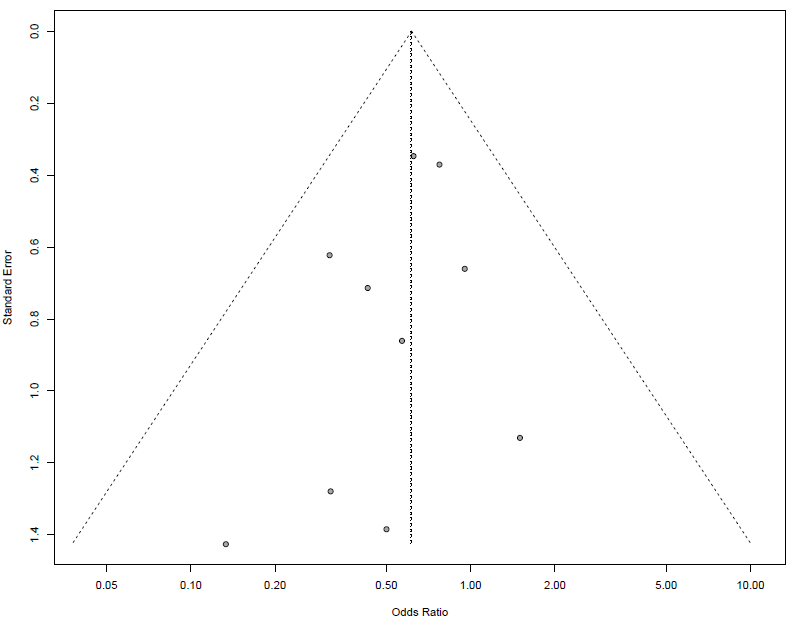


**E**

**Supplementary Figure 2. Funnel plots of meta-analysis on associations of hyperprogressive disease with serum lactate dehydrogenase (LDH) above/within the normal range (A); More**

**than /no more than two metastatic sites (B); Eastern Cooperative Oncology Group (ECOG) score ≥2/<2 (C); Liver metastases/No liver metastasis (D); Positivity/Negativity tumor programmed death 1 ligand 1 (PD-L1) expression status (E).**


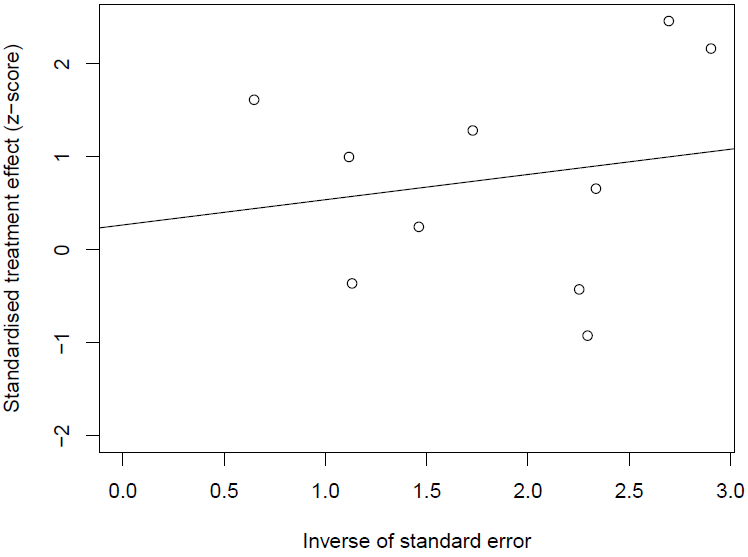


**A**


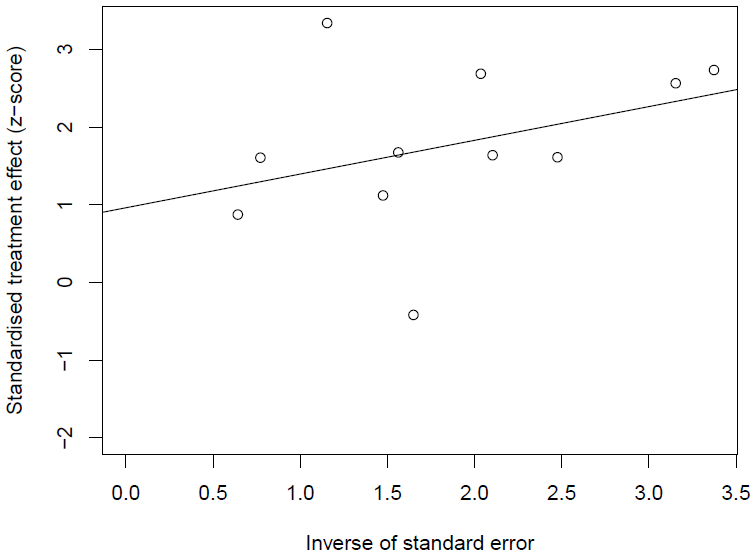


**B**


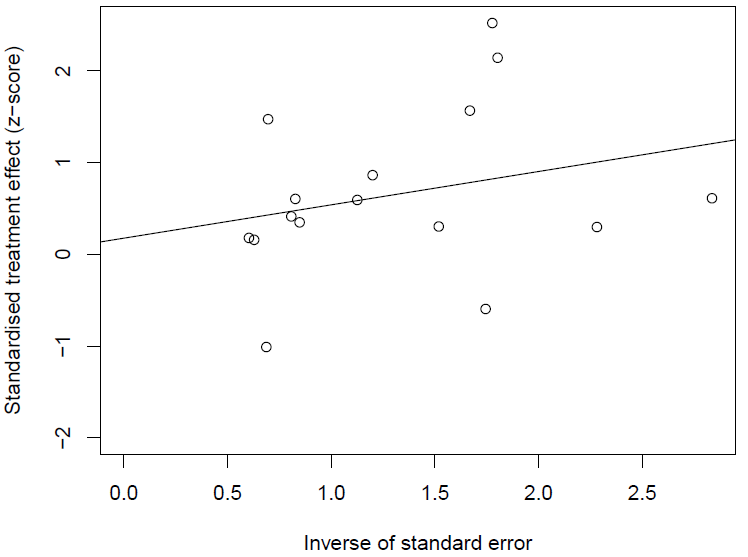


**C**


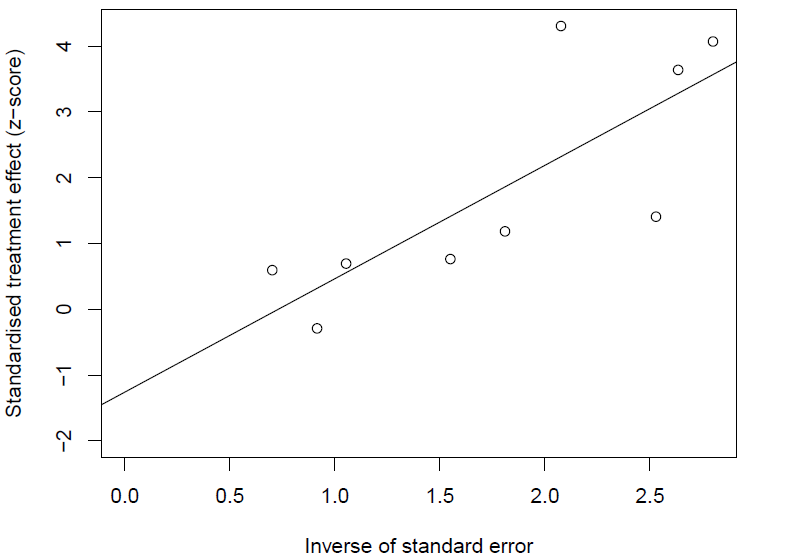


**D**


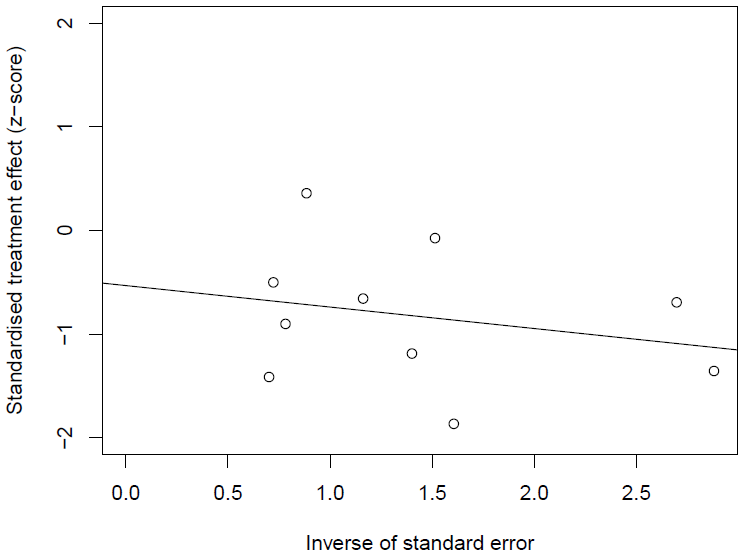


**E**

**Supplementary Figure 3. Plots of Egger’s tests illustrating publication bias for meta-analysis on associations of hyperprogressive disease with serum lactate dehydrogenase (LDH) above/within the normal range (A); More than /no more than two metastatic sites (B); Eastern Cooperative Oncology Group (ECOG) score ≥2/<2 (C); Liver metastases/No liver metastasis (D); Positivity/Negativity tumor programmed death 1 ligand 1 (PD-L1) expression status (E).**
